# Supplementary material for: An engineered bacterial symbiont maps micron-scale sugar gradients in the honeybee gut
Source: PLoS Biol. 2026 Sep 16;24(9):e3003997. doi: 10.1371/journal.pbio.3003997 (PMC13608000; doi:10.1371/journal.pbio.3003997)
Supplement: Supplementary file 1 [file pbio.3003997.s018.pdf]

REVIEWS:

Authors: We thank all four reviewers for their careful evaluation of our manuscript, as well as their constructive feedback. We have now addressed the reviewer's comments. Please find a detailed point-by-point reply below. We feel the changes have improved the manuscript and hope the reviewers agree.

Reviewer #1: In this study, Chhun et al. present a biosensor designed to provide insight into spatial gradients of arabinose metabolism. Using newly characterised promoters, the authors demonstrate the successful domestication of a non-native arabinose-inducible circuit for functionality in Snodgrassella alvi. The dynamic range of the system was fine-tuned by modulating expression levels of the genes araE and araC through newly screened promoters, as well as by altering plasmid backbones to control the copy number of either the araC transcription factor or the regulated GFP reporter. Building on this design, the authors demonstrate that the biosensor circuit remains stable even in the absence of selection and exhibits strong specificity, with induction occurring exclusively in response to arabinose. Finally, they showcase the utility of the biosensor in resolving both longitudinal and transversal spatial variation in arabinose concentrations. This is achieved by deploying the S. alvi biosensor in mono-colonised bees or in co-colonisation with different Gilliamella species, under conditions where bees were fed either pure arabinose or pollen-derived arabinose.

This work represents a significant advance over existing inducible genetic circuits in S. alvi, which have largely relied on the non-physiologically relevant inducer IPTG. By contrast, this study demonstrates that a biosensor can be used to monitor biologically relevant sugar metabolites in a spatially resolved manner. Overall, the study highlights the power of in vivo gut biosensors and is likely to inspire future efforts to engineer additional biosensors in other honeybee gut symbionts, enabling deeper insight into key metabolic processes within the honeybee gut microbiome and their broader implications for honeybee holobiont physiology. This should be of interest to the readership of PLoS Biology who will appreciate the coming together of microbiology, molecular biology, insect biology, bioengineering and environmental research that this study represents. It is high quality interdisciplinary work that should interest a broad readership.

Below are a list of minor comments which need to be addressed:

1. In numerous figures, some of the text has a red error line which normally indicates a spelling error. This is seen in Fig 1a - "sensing", Fig 3b - "Merged" and "Brightfield", Fig 4a - "biosensor", "S. alvi" and "days", "G. apicola, "Colonization", "Cup-housed", "gut", "analysis" and "days", Fig 4b - "S. alvi and "G. apicola". Please correct this in the figures.

Authors: We thank the reviewer for spotting this. We have modified the figures accordingly.

2. The dose response curve in Fig 1c shows the fold-change activity at the lowest non-inducing concentration relative to the uninduced conditions across the various designs is 1.4-fold. It would be wise to comment on, perhaps in the discussion, as to how this signal could be boosted to improve the signal to noise ratio further (maybe through amplification circuits?). In addition, please also refer to Armbruster and Pry (2008) (https://pmc.ncbi.nlm.nih.gov/articles/PMC2556583/) to determine the true detection limit of

the S. alvi biosensor which needs to be reported, especially given that the smaller reported induction values of 1.1-1.4-fold may just be within the analytical noise of the biosensor.

Authors: We now report the detection limit of the strain:

lines 143-144: "*The detection limit of this strain was 6.7 µM arabinose, corresponding to a 1.7-fold increase in fluorescence signal (Welch's t-test,* t*(4.6) = 14.8,* p*-value < 0.0001).*"

We also discuss potential ways to improve the signal to noise ratio further:

lines 364-369: "*We demonstrated that our biosensor can detect a biologically relevant range of arabinose concentrations with sufficient sensitivity. For applications requiring detection of lower concentrations, however, future iterations of the biosensor could increase sensitivity, for example, by incorporating a genetic amplification circuit[52]. At the same time, such enhancements would need to be balanced with maintaining stable and reliable performance* in vivo.*"*.

3. In all diagrams which depict genetic circuits, the promoters have been annotated, but other key modular parts such as RBSs or terminators have not been annotated. These should be corrected.

Authors: We thank the reviewer for this suggestion. The genetic parts used in our constructs, including RBSs and terminators, are not novel elements but are derived from a previously published toolkit for bee gut symbionts (see Leonard *et al*., 2018). As such, our figures were designed to highlight the new and key regulatory features relevant to the biosensor function, particularly promoters and overall plasmid architecture. This allows us to keep the figures clear and easy to read.

To ensure full transparency and reproducibility, we provide complete plasmid maps and sequences for all constructs on Addgene (see **lines 714-718**), where all modular elements, including RBSs and terminators, are fully annotated and readily accessible. We now refer to this point in the **caption of Figure 1a**: "*For clarity, certain regulatory elements have been omitted from the diagram. Complete annotations and sequence details are available in the Addgene repository.*"

We believe this approach maintains clarity in the figures while allowing interested readers to access detailed construct information.

4. In Supplementary Fig. 3c, the images depicting an overview of the biosensor strains are quite pixelated. This should be replaced with a higher quality version.

Authors: We have replaced the pixelated images.

5. The authors present newly characterized promoters for S. alvi in Supplementary Fig. 1. I suggest incorporating this figure as an additional subpanel within Fig. 1. Doing so would better justify the selection of specific promoters used for circuit design and make these genetic parts more visible and accessible to readers interested in applying them in future work. There appears to be sufficient space in Fig. 1 to accommodate this addition. In addition, Supplementary Fig. 3a-b could be integrated as further subpanels in Fig. 1 to more clearly illustrate the engineering strategy used to generate a functional arabinose-inducible promoter. Specifically, it would be valuable to include colony images for all pBAD designs, rather than only the single uninduced and induced examples currently shown in Fig. 1b. The domestication of inducible promoters

for use in non-model organisms is technically challenging, and the substantial effort undertaken here to establish a functional system in S. alvi merits more prominent presentation in the main figures. The text accompanying Fig. 1 should be revised accordingly to reflect these additions and to explicitly highlight the rationale for promoter selection as well as the iterative design process underlying the development of the inducible system.

Authors: We thank the reviewer for recognizing the technical challenges associated with the development of inducible systems in non-model organisms and for appreciating the importance of this aspect of our work.

We have modified the **Figure 1** to reflect the reviewer's suggestions. Figure 1 now contains the characterization of the promoter (previously Supplementary Fig. 1) as the subpanel b; the sequence of the pBAD25 promoter (previously Supplementary Fig. 3a) as the subpanel c and the colony images for the pBAD25 design (previously Supplementary Fig. 3b) as the subpanel d.

6. Lines 166 to 169: "While part of the biosensor design was chromosomally integrated, we hypothesized that plasmid-borne elements might be lost under non-selective conditions, as expected in the natural gut environment". If stable genome integration is achievable for parts of the biosensing circuit, such as the araE gene which is integrated at the tetC locus shown in this work, could other components of the circuit also not have been genome integrated to enhance the stability of the circuits (i.e the araC gene and the reporter construct) - either at the same locus at araE or at different loci? Please comment.

Authors: We thank the reviewer for this insightful suggestion.

In principle, full chromosomal integration of the biosensing circuit could indeed improve genetic stability. However, in *S. alvi*, the number of well-characterized and reliably neutral genomic integration sites ("landing pads") is currently limited. In our hands, while we explored several loci, the *tetC* locus was the only site that consistently allowed efficient integration without detectable fitness or expression side effects.

We also considered integrating additional components of the circuit at the same locus. However, we found that the efficiency of genomic integration decreased substantially with increasing fragment size, making the insertion of larger, multi-gene constructs technically challenging. This limitation arises both from reduced efficiency of recombination and from the practical constraints of amplifying large homologous linear recombination fragments.

In contrast, maintaining parts of the circuit on plasmids provided important flexibility during the design phase, allowing us to rapidly test and optimize different configurations. We also found that plasmid-based expression supported robust performance of the inducible system, whereas full genomic integration could potentially reduce expression levels and thus impact the dynamic range and sensitivity of the biosensor.

We agree that further development of additional genomic integration sites in *S. alvi* would be highly valuable for future work aimed at fully chromosomally encoded circuits.

We added the following text to the discussion:

Lines 343-355: "*By combining chromosomal integration with a suite of low-strength promoters, we overcome these limitations, achieving stable and predictable multi-gene expression in* S. alvi *without compromising its capacity to colonize the gut, albeit within a restricted but experimentally relevant time window. The number of well-characterized and reliably neutral genomic integration sites (*i.e. *landing pads) in* S. alvi *remains, however,*

limited. Expanding the repertoire of defined landing pads would facilitate the chromosomal encoding of increasingly complex genetic circuits, reducing reliance on plasmids and further enhancing the long-term stability of engineered strains. Nevertheless, the expanded genetic control presented here enhances the tractability of S. alvi *and broadens its potential as a chassis for in vivo studies and microbiome engineering. Extending beyond our previous work with synthetic inducers, it now allows the implementation of more complex functionalities for nutrient biosensing within the gut environment.*"

7. Lines 160-165: "Using this normalization for the gut volume, arabinose concentrations averaged 2.2 ± 1.1 mM in MD bee guts, and 0.0722 ± 0.0164 mM in CB bee guts (n = 8; Fig. 2b). This range falls within the detection window of the engineered S. alvi strain ac02 (Fig. 1c), suggesting it can report variations in arabinose concentrations of both extremes: high concentrations in the absence of microbial metabolism (i.e. MD bees) and reduced, ecologically relevant levels when the native gut community consumes arabinose (i.e. CB bees)." ◊ In this section, can the claim "ecologically relevant levels when the native gut community consumes arabinose" be true? This experimental set up does not comprise a complete honeybee native gut microbiome community and therefore technically not ecologically relevant in the context of the experiment conducted. In which case, the sentence should be revised to reflect this.

Authors: Please find our consolidated response to points 7 and 8 below.

8. As a comment which follows on from point 7, the authors mention "previous studies have identified Gilliamella and Bifidobacterium as the principal degraders of complex carbohydrates in the bee gut" - lines 60 to 61. How different would the experimentally determined arabinose concentrations (0.0722 mM) be in the presence of other microbiome members which also metabolise complex carbohydrates such as arabinose? If more microbiome members are metabolising carbohydrates, I would suspect that the levels may end up below the detection range of the biosensor. Would this not impact the utility of an arabinose biosensor under a complete microbiome community? It would be wise to make a comment on this in the text.

Authors: We thank the reviewer for raising this important point and we apologize for not providing a more detailed description of the synthetic community in the manuscript. We would like to clarify that the colonized bees (CL) were associated with a defined bacterial community comprising 25 isolates representing the most prevalent and abundant members of the honey bee gut microbiota. The community notably includes multiple strains of *Gilliamella* (n = 5) and *Bifidobacterium* (n = 8), which are key contributors to carbohydrate metabolism in the bee gut.

The design of this community is part of a distinct line of work in our group, but it was informed by extensive metagenomic analyses of the honey bee gut microbiota. While these data are not yet published and therefore cannot be formally cited, they guided the selection of strains to capture dominant and functionally relevant members of the community.

To address the reviewer's concern, we have revised the manuscript to avoid overgeneralization, toning down the use of the term "native gut community" and "ecologically relevant levels":

lines 180-184: "*This range falls within the detection window of the engineered* S. alvi *strain ac02 [...] and reduced levels when a gut community consumes arabinose (*i.e. *CL bees)*"

and provided some clarification both in the manuscript and in the Methods sections.

Lines 171-173: *"To determine these levels, we dissected guts from pollen-fed, microbiota-deprived bees (MD) or individuals colonized by a defined bacterial community of 25 isolates (CL, see details in the methods),"*

lines 574-578: "*Arabinose levels in the gut were compared between MD bees and those colonized with a synthetic community of 25 isolates that encompass highly prevalent and abundant species within the gut microbiota of honeybees comprising the* Bartonella*,* Bifidobacterium*,* Bombilactobacillus*,* Commensalibacter*,* Frischella*,* Gilliamella*,* Lactobacillus *and* Snodgrassella *genera.*".

Furthermore, while this defined community does not fully capture the complexity of the gut microbiota of conventional honey bees, previous work has shown that smaller synthetic communities can recapitulate metabolic phenotypes of wild bees (see Kesnerová et al., "*Disentangling metabolic functions of bacteria in the honey bee gut*", 2017, DOI: https://doi.org/10.1371/journal.pbio.2003467). Therefore, we expect that the arabinose levels measured in here reflect substantial microbial degradation and are representative of physiologically relevant conditions, albeit being subject to the usual caveats of bees raised in laboratory environments *versus* in the wild.

We agree, however, that further metabolic activity is a possibility with a more complete microbiome community, which could reduce arabinose concentrations beyond those measured here. This could, in principle, bring concentrations closer to or below the lower detection range of the biosensor. However, our results indicate that the biosensor can detect concentrations an order of magnitude lower than those observed with this synthetic community. We have nevertheless amended the discussion to acknowledge potential limitations in sensitivity of the biosensor and to more broadly discuss opportunities for further optimization of the biosensor:

lines 364-369: "*We demonstrated that our biosensor can detect a biologically relevant range of arabinose concentrations with sufficient sensitivity. For applications requiring detection of lower concentrations, however, future iterations of the biosensor could increase sensitivity, for example, by incorporating a genetic amplification circuit*[48]*. At the same time, such enhancements would need to be balanced with maintaining stable and reliable performance in vivo*."

9. The authors report values of "2.2 ± 1.1 mM in MD bee guts, and 0.0722 ± 0.0164 mM in CB bee guts", please convert these to µM values so that it makes it easier to infer where they are on dose response curve of biosensor S. alvi ac02 (Fig. 1c) where the x-axis units are µM.

Authors: We have changed the unit according to the reviewer's suggestion (see **lines 179-180**).

10. Figure 2c investigates the stability of the genetic circuit in liquid culture in the absence and presence of antibiotic selection. As the paper focuses on the utility of a biosensor for in vivo monitoring, is this figure relevant in the context of the rest of the paper considering it is an experiment not representative of the gut environment?

Authors: Figure 2c provides an initial assessment of biosensor stability, showing that the genetic construct imposes a measurable but relatively modest metabolic burden *in vitro*, as indicated by partial plasmid loss without antibiotic selection. In contrast, some strain designs we previously tested for other biosensors showed significant instability *in vitro* such that we could not move on to *in vivo* testing. In our opinion, the *in vitro* results shown in Figure 2c are thus crucial, and positive, as they encouraged us to pursue with this construct to the *in vivo* experiments (**Figure 3**).

11. In relation to Fig 3a which demonstrates the bacterial load in the absence and presence of antibiotic selection, could a similar style of experiment not have been performed to assess plasmid stability at day 7 and 14 in vivo?

Authors: Reviewers 2 and 4 raised similar concerns. To address them, we performed additional experiments, as suggested by the reviewers, to investigate plasmid maintenance during colonization of the bee gut. Briefly, we conducted an *in vivo* experiment similar to that shown in **Figure 3a**. In this case, fecal samples were additionally plated on appropriate selective media to quantify plasmid maintenance in the biosensor cells (as in **Figure 2c** and **Supplementary Figure 4**) following colonization of bees that were fed either with or without antibiotic supplementation. Plasmid maintenance was assessed after 7 and 14 days.

The resulting data, presented in the new **Supplementary Figure 5a**, are consistent with our *in vitro* observations. Specifically, plasmid pAC51c was maintained at high levels (75.3 ± 23.5 %) over the two-week period, whereas plasmid pAC51b showed lower stability in the absence of antibiotic selection in the gut. Notably, pAC51b retention was similar *in vitro* (**Supplementary Figure 4a)** and *in vivo* (**Supplementary Figure 5a)**, averaging 37.8 ± 6.9 % and 33.5 ± 27.6 %, respectively (Mann-Whitney U test, p-value = 0.198).

In addition, to verify that cells retaining the plasmids remained functional and had not accumulated mutations compromising biosensor activity, we tested GFP inducibility in a subset of colonies randomly selected from the day 7 CFU-counting experiment. Colonies were recovered from antibiotic-selective plates, ensuring retention of both plasmids, and originated from bees fed either with or without antibiotic supplementation. As shown in the new **Supplementary Figure 5b**, cells recovered from both *in vivo* conditions retained robust responsiveness to arabinose and exhibited expected GFP induction. These results indicate that our biosensor remained functional throughout gut colonization and are consistent with the findings presented throughout the manuscript (**Figures 3 and 4**).

To describe these experiments, we also added the following text to the manuscript:

Lines 228-240: "*To determine whether the biosensor remained genetically stable during gut colonization, we additionally quantified plasmid maintenance* in vivo *by plating fecal samples from bees fed with or without antibiotic supplementation on selective media. Consistent with our* in vitro *observations, the gfp-bearing plasmid pAC51c was maintained at high levels throughout the two-week experiment (75.3 ± 23.5 % without antibiotic selection), whereas maintenance of the araC-bearing plasmid pAC51b declined in the absence of antibiotic selection (**Supplementary Fig. 5a**). Notably, pAC51b retention was similar* in vitro *and* in vivo*, averaging 37.8 ± 6.9 % and 33.5 ± 27.6 %, respectively (Mann-Whitney U test,* p-value *= 0.198). To assess whether plasmid-bearing cells remained functional, we further tested arabinose-induced GFP expression in colonies recovered from the same colonized bees at day 7. Cells isolated from bees fed both antibiotic-supplemented and normal sugar water retained robust inducibility (**Supplementary Fig. 5b**), indicating that the biosensor remained functional throughout gut colonization over the time frame examined.*"

12. Figure 3b caption: "A confocal microscopy image of a honeybee gut colonized by engineered S. alvi ac02 in the ileum region. A bacterial biofilm within a gut crypt is shown. The blue channel depicts DAPI staining of host and bacterial DNA and the green channel shows GFP fluorescence from the engineered strain". Whie in the main body of the text it is mentioned

that Fig 3b depicts basal GFP levels in the absence of induction, please make it clear in the caption that this is in the absence of arabinose.

Authors: We have now amended the figure caption of Figure 3b to include the information mentioned: "*The bee was fed sugar water without arabinose supplementation*."

Reviewer #2: The authors engineer S. alvi as a living biosensor for arabinose in the honeybee gut, achieving micron-scale spatial resolution of sugar gradients within bacterial biofilms. The paper advances the field on two fronts: expanding the genetic toolkit for a non-model symbiont and applying it to reveal previously inaccessible nutrient microenvironments in vivo. The work is well-suited for PLOS Biology. However, there is a minor issue that should be addressed.

Plasmid stability. Supplementary Figure 5a indicates that the araC plasmid does not achieve 100% maintenance even under antibiotic selection. What might explain this incomplete retention?

Authors: We hypothesize that the incomplete retention mentioned by the reviewer results from the use of ampicillin as a selective agent for the AraC plasmid. Ampicillin resistance relies on β-lactamase activity, which can degrade the antibiotic in the surrounding medium and thereby reduce selective pressure over time. During a prolonged 3-day liquid culture, this may in turn allow a limited proportion of cells to lose their plasmids and divide once the selection pressure has been sufficiently attenuated, resulting in a heterogenous population of plasmid-free and plasmid-bearing cells. The population subsequently plated onto non-selective media thus may include a fraction of plasmid-free cells, leading to an apparent reduction in plasmid maintenance even in the "+Ab" condition. This phenomenon has previously been well documented, as β-lactamase-producing cells can detoxify the environment and allow the growth of plasmid-free "cheater" cells, resulting in mixed populations despite antibiotic selection (see Bedore & Waters, "*Plasmid-free cheater cells commonly evolve during laboratory growth*", 2024, DOI: https://doi.org/10.1128/aem.02311-23).

We now comment on this point in the figure caption of **Supplementary Figure 4** (due to a change of the number): "*Incomplete maintenance of pAC51b in the + ab condition likely reflects the use of ampicillin selection, as β-lactamase-mediated degradation of ampicillin can reduce selection pressure during prolonged liquid culture and permit the expansion of plasmid-free cells.*"

In addition, plasmid stability was assessed only after 3 days in vitro (Fig. 2c), whereas in vivo imaging was performed at day 7. Plasmid maintenance at this later time point, particularly in the absence of antibiotic selection, could be substantially lower. Since Figure 3a already involves selective plating of fecal samples, performing parallel plating on single-antibiotic and no-antibiotic plates would enable calculation of in vivo plasmid retention at day 7, which would strengthen the analysis.

Authors: Reviewers 2 and 4 raised similar concerns. To address them, we performed additional experiments, as suggested by the reviewers, to investigate plasmid maintenance during colonization of the bee gut. Briefly, we conducted an *in vivo* experiment similar to that shown in **Figure 3a**. In this case, fecal samples were additionally plated on appropriate selective media to quantify plasmid maintenance in the biosensor cells (as in **Figure 2c** and **Supplementary Figure 4**) following colonization of bees that were fed either with or without antibiotic supplementation. Plasmid maintenance was assessed after 7 and 14 days.

The resulting data, presented in the new **Supplementary Figure 5a**, are consistent with our *in vitro* observations. Specifically, plasmid pAC51c was maintained at high levels (75.3 ± 23.5 %) over the two-week period, whereas plasmid pAC51b showed lower stability in the absence of antibiotic selection in the gut. Notably, pAC51b retention was similar *in vitro*

(**Supplementary Figure 4a)** and *in vivo* (**Supplementary Figure 5a)**, averaging 37.8 ± 6.9 % and 33.5 ± 27.6 %, respectively (Mann-Whitney U test, p-value = 0.198).

In addition, to verify that cells retaining the plasmids remained functional and had not accumulated mutations compromising biosensor activity, we tested GFP inducibility in a subset of colonies randomly selected from the day 7 CFU-counting experiment. Colonies were recovered from antibiotic-selective plates, ensuring retention of both plasmids, and originated from bees fed either with or without antibiotic supplementation. As shown in the new **Supplementary Figure 5b**, cells recovered from both *in vivo* conditions retained robust responsiveness to arabinose and exhibited expected GFP induction. These results indicate that our biosensor remained functional throughout gut colonization within this time window and suggests that the accumulation of non-functional mutants is limited under these conditions, consistent with the findings presented throughout the manuscript (**Figures 3 and 4**).

To describe these experiments, we also added the following text to the manuscript:

Lines 228-240: "*To determine whether the biosensor remained genetically stable during gut colonization, we additionally quantified plasmid maintenance* in vivo *by plating fecal samples from bees fed with or without antibiotic supplementation on selective media. Consistent with our* in vitro *observations, the gfp-bearing plasmid pAC51c was maintained at high levels throughout the two-week experiment (75.3 ± 23.5 % without antibiotic selection), whereas maintenance of the araC-bearing plasmid pAC51b declined in the absence of antibiotic selection (**Supplementary Fig. 5a**). Notably, pAC51b retention was similar* in vitro *and* in vivo*, averaging 37.8 ± 6.9 % and 33.5 ± 27.6 %, respectively (Mann-Whitney U test,* p-value = *0.198). To assess whether plasmid-bearing cells remained functional, we further tested arabinose-induced GFP expression in colonies recovered from the same colonized bees at day 7. Cells isolated from bees fed both antibiotic-supplemented and normal sugar water retained robust inducibility (**Supplementary Fig. 5b**), indicating that the biosensor remained functional throughout gut colonization over the time frame examined.*"

However, we acknowledge that stability at later time points *in vivo* was not assessed and may represent a limitation. We have now clarified this point in the Discussion as a potential source of variability in longer-term applications:

lines 345-347: "*… achieving stable and predictable multi-gene expression in S. alvi without compromising its capacity to colonize the gut, albeit within a limited but experimentally relevant time window.*";

lines 349-354: "*While our data indicate that the biosensor remains functional within a 7-day experimental window, the genetic stability of the construct over longer time scales was not directly assessed, and the potential accumulation of non-functional mutations or plasmid loss at later time points may influence its performance for longer term measurements. Future work could address this limitation by quantifying functional stability over more extended time periods and by implementing more stable genetic designs.")*.

We also added details about the timeline of the experiments in the result and the methods section, as well as the figure legends:

lines 211-213: "*… we focused on whether the engineered strain could establish in the honeybee gut, how long it could persist and whether it could sense arabinose in situ and provide spatial information on sugar availability*";

lines 225-226: "*Based on these observations, all subsequent measurements were performed 7 days post-colonization*";

lines 244-245: "*After 7 days, we dissected guts and imaged them by confocal microscopy*";

lines 663-665: "*… honeybee hindguts were collected seven days after inoculation with engineered S. alvi and Gilliamella, a time point selected to ensure sufficient biosensor abundance for reliable sugar detection, and …*";

Figure 3 legend; **Figure 4 legend**

Reviewer #3: This exciting and solid work demonstrates a simple biosensor and its utility in the honeybee gut. While relatively well-designed, this work would be stronger if the following points were addressed.

1. Surprisingly, many key reports on biosensors developed for potential applications in microbiota are missing, including, but not limited to, those from the laboratory of Pam Silver, Jeff Tabor, Tim Lu, Tae Seok Moon, Harris Wang, Matthew Chang et al. In addition, key reports on the bee gut microbiome are missing, including, but not limited to, that from the Jeffrey Barrick lab. The authors should perform extensive literature search to cite and discuss relevant previous work.

Authors: We acknowledge that there is extensive and important work on biosensors in the literature. Due to space constraints and limits on the number of citations, we initially focused on studies most relevant to insect systems and sugar sensing, and therefore included fewer references related to the human gut microbiota. Following the reviewer's suggestion, we have now revised the introduction to incorporate additional relevant biosensor literature, *i.e.* references **28-31**.

2. I understand the challenges of developing biosensors in non-model organisms. However, the choice of sugar arabinose reduces my enthusiasm. I am less convinced regarding the utility and novelty of this work due to this choice and given that the authors developed another biosensor for IPTG in their previous report. Still, implementing this biosensor in the bee gut would be novel, but I would recommend a simple demonstration (not extensive though) of a third, more useful sensor in the bee gut because the proof-of-concept demonstration was made with the IPTG sensor.

Authors: We appreciate that the reviewer recognizes the challenges inherent in working with non-model organisms and we would like to provide further context regarding our choice to report arabinose sensing only in this study.

Regarding the choice of arabinose, we understand the reviewer's perception that arabinose may appear less novel given the widespread use of the AraC–pBAD system in molecular and synthetic biology. However, we would like to emphasize that implementing this sensing capability in *S. alvi* required substantial engineering efforts. These included establishing functional arabinose uptake, developing and characterizing new genetic parts, improving genomic integration strategies, and designing a synthetic promoter responsive to arabinose in this non-model organism. Thus, the novelty of our work lies not only in the sensing module itself, but in enabling its functionality in a previously intractable host and deploying it *in vivo*.

More importantly, arabinose is a *bona fide* biologically significant component of the honey bee gut. It is a natural part of the bee diet and serves as a key substrate for some members of the microbiota, such as *Gilliamella*, whose metabolism generates organic acids that are cross-fed to *S. alvi*. In contrast to our previous work using IPTG as a synthetic inducer, the present study moves beyond a proof-of-principle demonstration by targeting a naturally occurring dietary compound and revealing its spatial distribution and microbial utilization *in vivo*. We consider that this biosensor provides meaningful insight into nutrient availability and microbial interactions in the gut environment.

We agree that expanding the range of biosensors in *S. alvi* will enhance the platform's applicability. To this end, we are currently developing additional sensors targeting other biologically relevant molecules in the honey bee gut, including oxygen and N-acetylglucosamine, for which we have obtained promising *in vitro* results demonstrating

sensing capabilities in engineered *S. alvi* strains. However, establishing these systems for robust *in vivo* application still requires substantial optimization and validation work, that will take place over extended timeframes. Accordingly, it would extend beyond the scope of the current revision. Moreover, these systems involve distinct molecular mechanisms and are intended to address different biological questions, and will thus be presented in separate manuscripts.

3. When comparing data, I suggest that authors should use statistical analysis with p-value reported in the figure caption and figures if possible.

Authors: We thank the reviewer for this suggestion. Statistical analyses are included in our figures using a lettering scheme to indicate significant differences between groups. We chose this format to maintain clarity. The statistical details are provided in the figure captions, where we specify that different letters indicate significant differences (*i.e.* corresponding to a given p-value that we provide in the corresponding figure caption). We believe this approach ensures both readability and appropriate reporting of statistical significance.

In response to the reviewer's comment, we have added supplementary statistical analyses to provide formal statistical support for reported findings where comparisons between experimental conditions were especially important (*i.e.* **Figures 3e and 4d**).

Statistical information was added in the corresponding Figure captions and in the main text:

lines 312-318:"*The biosensor reported fluorescence intensities consistent with the arabinose concentrations fed to the host but exhibited no longitudinal variation, with similar fluorescence levels across all three regions for a given sugar condition (**Fig. 3e**; two-way ANOVA, effect of ileum section not significant: F(2, 95) = 0.03, p-value = 0.972). These results indicate that arabinose is homogeneously distributed along the ileum's length.*"

lines **299-303**: "*which were notably independent of the Gilliamella species present (Fig. 4d; three-way ANOVA, effect of species not significant: F(1, 90) = 0.19, p-value = 0.662). Also, fluorescence measurements of* S. alvi *along the ileum's length revealed highly heterogenous arabinose concentrations between bees, although homogeneously distributed longitudinally (**Fig. 4d;** three-way ANOVA, effect of ileum section not significant: F(2, 90) = 0.44, p-value = 0.648) and overall consistent within each individual (**Supplementary Fig. 8**).*"

4. Given that biosensors have been developed for microbiome applications for more than a decade, the authors need to tone down, minimizing overclaiming.

Authors: We fully agree that biosensors for microbiome applications have been developed and refined over many years (references **28-32**). However, most of this work has been carried out for biosensing in the environment, in mammalian hosts and some select invertebrates[24,25]. To the best of our knowledge, they have not been applied to insect models.

We have aimed to present our work in a balanced and accurate manner. To this end, we have carefully re-evaluated the manuscript and made the revisions outlined in this rebuttal to ensure that all claims are appropriately supported by the data. If the reviewer feels that any statements are still overclaimed, we would greatly appreciate more specific guidance on the relevant sections so that we can address them directly.

5. Readers would appreciate it if the authors discuss the limitations of their approach using this sensor in more details.

Authors: We have revised the manuscript to better discuss the limitations of our approach. Specifically, we expanded the discussion to address limitations in sensitivity and long-term *in vivo* genetic stability. These points are now explicitly discussed in the revised manuscript:

lines 349-369: "*While our data indicate that the biosensor remains functional within a 7-day experimental window, the genetic stability of the construct over longer time scales was not directly assessed, and the potential accumulation of non-functional mutations or plasmid loss at later time points may influence its performance for longer term measurements. Future work could address this limitation by quantifying functional stability over more extended time periods and by implementing more stable genetic designs. The number of well-characterized and reliably neutral genomic integration sites (*i.e. *landing pads) in* S. alvi *remains, however, limited. Expanding the repertoire of defined landing pads would facilitate the chromosomal encoding of increasingly complex genetic circuits, reducing reliance on plasmids and further enhancing the long-term stability of engineered strains.*

Nevertheless, the expanded genetic control presented here enhances the tractability of S. alvi *and broadens its potential as a chassis for* in vivo *studies and microbiome engineering. Extending beyond our previous work with synthetic inducers, it now allows the implementation of more complex functionalities for nutrient biosensing within the gut environment.*

We demonstrated that our biosensor can detect a biologically relevant range of arabinose concentrations with sufficient sensitivity. For applications requiring detection of lower concentrations, however, future iterations of the biosensor could increase sensitivity, for example, by incorporating a genetic amplification circuit[52]*. At the same time, such enhancements would need to be balanced with maintaining stable and reliable performance* in vivo."

Reviewer #4: In this manuscript the authors create an arabinose "in vivo biosensor" strain of S. alvi, expanding their previous work demonstrating IPTG induction in this bee gut associated bacterium. They demonstrate chromosomal integration of the araE transporter as well as a two-plasmid sense and respond circuit that expresses GFP in response to the presence of arabinose in a dose dependent manner. The authors then test this circuit's function in the context of the bee gut and the presence or absence of pollen and co-colonizing G. apicola strains. The authors report on potential arabinose gradients in the bee gut and identify highly variable arabinose-rich micro-environments as the result of pollen degradation. They propose the biosensor as a highly sensitive and spatially resolved reporter of arabinose concentration in vivo. Overall, this work is an appropriate and interesting update to the initial report. I welcome the expanded engineering approaches in bee gut bacteria and the use of engineered microbes as sensitive biosensors for complex and inaccessible gut environs. I have several significant concerns, however, which may undermine the reported findings unless they are addressed. To reliably trust the biosensor output the authors should strongly demonstrate the sensor is specific to arabinose, is genetically stable, and has a high dynamic range both in vitro and in vivo.

Major concerns:

-The characterization of the biosensor's arabinose specificity is insufficient. As the authors correctly point out, the bee gut is a complex environment rich in carbohydrates. Testing alternative sugars at a single concentration as in Figure 2a is a good first step. However, sugar concentration impacts sensor function and araE can import alternative sugars, possibly impacting S. alvi physiology. To verify specificity, the authors should further characterize the biosensor in the laboratory across a range of concentrations of ribose and xylose, and test 50:50 mixtures of arabinose with alternative sugars. Ideally, the authors would also demonstrate that the biosensor is not activated in bees colonized with ac02 and fed ribose or xylose. However, this experiment is substantial work and this caveat about specificity in vivo could instead be addressed in the discussion.

Authors: We thank the reviewer for raising this important point regarding the biosensor specificity.

It is correct that AraE is a low-affinity transporter that can mediate limited uptake of other sugars under certain conditions. However, we would like to emphasize that transport does not determine the specificity of the system. Rather, specificity is governed at the regulatory level by AraC.

To address the reviewer's concern, we performed complementary *in vitro* experiments in which the biosensor strain was incubated in media supplemented with 0.7 mM ribose, xylose, or arabinose, as well as equimolar mixtures of ribose/arabinose and xylose/arabinose. Fluorescence measurements (**see updated Figure 2a**) demonstrated that the biosensor retained its specificity for arabinose, showing robust induction only in the presence of arabinose and no detectable response to ribose or xylose.

This aligns with established literature describing AraC as a well-characterized, ligand-specific transcription factor whose activation strictly depends on binding L-arabinose (Schleif, 2010, DOI:10.1111/j.1574-6976.2010.00226.x). Extensive genetic, biochemical, and structural studies have previously demonstrated that structurally and chemically similar sugars such as glucose, sucrose, glycerol, fucose, xylose, or ribose do not induce the AraC–pBAD system (Kang et al., 1998, DOI: 10.1016/S1016-8478(23)13430-3; Doyle et al., 1972, DOI: 10.1128/jb.110.1.56-65.1972; Ross et al., 2003, DOI:10.1016/S0022-2836(03)00262-6).

Instead, AraC exhibits such stringent specificity that substantial protein engineering is required to enable responsiveness to D-arabinose instead of L-arabinose, but even those AraC variants were shown to not respond to D-xylose, D-fucose nor D-lyxose (Tang et al., 2008, DOI: 10.1021/ja7109053). These studies were added as **new references 47-49**.

Finally, concentrations of the other pentose sugars xylose and ribose measured by GC-MS in the guts of pollen-fed colonized (now referred to as CL instead of CB for consistency with previous literature) bees during this study (data not shown in the manuscript) are similar to that of arabinose (*i.e.* Arabinose: 72.2 ± 16.4 µM; Ribose: 98.7 ± 29.5 µM; and Xylose: 114.4 ± 3.9 µM). Those levels of sugars are substantially lower than the one tested in our experiments (*i.e.* 700 µM). Therefore, even in the event of nonspecific transport, and would AraC be promiscuous to these sugars, their concentrations *in vivo* would remain below the levels at which we have already demonstrated no activation of the biosensor.

Taken together, both the literature and our experimental data support the conclusion that the observed response is specific to arabinose. We respectfully argue that this level of validation is therefore sufficient, and that additional specificity assays would be unlikely to yield further mechanistic insight beyond what is already well established in the literature. We have now amended the corresponding result section of the manuscript to reflect our reasoning supporting the specificity of our biosensor based on previous literature:

lines 164-168: "*Consistent with the well-documented specificity of the AraC-pBAD regulatory system for L-arabinose over related pentoses*[47-49]*, these results indicate that the engineered strain S. alvi ac02 provides a specific and reliable readout of arabinose in the presence of structurally related carbohydrates that are found in the bee diet.* ".

-I have concerns about the genetic stability of the biosensor construct and how that may impact the sensor's function in the bee gut. A biosensor construct that is too burdensome or prone to mutation is not a reliable reporter. Testing plasmid maintenance in vitro is an important but incomplete method to assess stability. For example, strains that accumulate non-functional mutations but maintain the plasmid are not captured in this assay but can be a substantial portion of cells, especially when constructs are burdensome. The fact that ~50% of colonies lose a plasmid in a single culture in the absence of antibiotic selection suggest substantial burden. The authors should repeat this stability assay and select colonies that maintain both plasmids and then test their function in the presence and absence of arabinose. As above, the strongest evidence would see the same stability demonstrated in bacterial colonies isolated after some period of colonization in honey bees from an experiment like that shown in Fig 3a. However, this could instead be addressed as a possible source of error in the discussion.

Authors: We appreciate the reviewer's concern with regards to the stability of the biosensor and agree this is an important point. Indeed, reviewers 1 and 2 raised similar concerns. Stability is critical for ensuring reliable reporting, and plasmid maintenance alone does not exclude the possibility of non-functional mutations.

To address the reviewers' concern, we performed additional experiments to investigate plasmid maintenance during colonization of the bee gut. Briefly, we conducted an *in vivo* experiment similar to that shown in **Figure 3a**. In this case, fecal samples were additionally plated on appropriate selective media to quantify plasmid maintenance in the biosensor cells (as in **Figure 2c** and **Supplementary Figure 4**) following colonization of individual bees that were fed either with or without antibiotic supplementation. Plasmid maintenance was assessed after 7 and 14 days.

The resulting data, presented in the new **Supplementary Figure 5a**, are consistent with our *in vitro* observations. Specifically, plasmid pAC51c was maintained at high levels (75.3 ± 23.5 %) over the two-week period, whereas plasmid pAC51b showed lower stability in the absence of antibiotic selection in the gut. Notably, pAC51b retention was similar *in vitro* (**Supplementary Figure 4a)** and *in vivo* (**Supplementary Figure 5a)**, averaging 37.8 ± 6.9 % and 33.5 ± 27.6 %, respectively (Mann-Whitney U test, p-value = 0.198).

In addition, to verify that cells retaining the plasmids remained functional and had not accumulated mutations compromising biosensor activity, we tested GFP inducibility in a subset of colonies randomly selected from the day 7 CFU-counting experiment. Colonies were recovered from antibiotic-selective plates, ensuring retention of both plasmids, and originated from bees fed either with or without antibiotic supplementation. As shown in the new **Supplementary Figure 5b**, cells recovered from both *in vivo* conditions retained robust responsiveness to arabinose and exhibited expected GFP induction. These results indicate that our biosensor remained functional throughout gut colonization within this time window and suggests that the accumulation of non-functional mutants is limited under these conditions, consistent with the findings presented throughout the manuscript (**Figures 3 and 4**).

To describe these experiments, we also added the following text to the manuscript:

Lines 228-240: "*To determine whether the biosensor remained genetically stable during gut colonization, we additionally quantified plasmid maintenance* in vivo *by plating fecal samples from bees fed with or without antibiotic supplementation on selective media. Consistent with our* in vitro *observations, the gfp-bearing plasmid pAC51c was maintained at high levels throughout the two-week experiment (75.3 ± 23.5 % without antibiotic selection), whereas maintenance of the araC-bearing plasmid pAC51b declined in the absence of antibiotic selection (**Supplementary Fig. 5a**). Notably, pAC51b retention was similar* in vitro *and* in vivo*, averaging 37.8 ± 6.9 % and 33.5 ± 27.6 %, respectively (Mann-Whitney U test,* p-value = *0.198). To assess whether plasmid-bearing cells remained functional, we further tested arabinose-induced GFP expression in colonies recovered from the same colonized bees at day 7. Cells isolated from bees fed both antibiotic-supplemented and normal sugar water retained robust inducibility (**Supplementary Fig. 5b**), indicating that the biosensor remained functional throughout gut colonization over the time frame examined.*"

We have also clarified the point of stability in the Discussion as a potential source of variability in longer-term applications:

lines 349-354: "*While our data indicate that the biosensor remains functional within a 7-day experimental window, the genetic stability of the construct over longer time scales was not directly assessed, and the potential accumulation of non-functional mutations or plasmid loss at later time points may influence its performance for longer term measurements. Future work could address this limitation by quantifying functional stability over more extended time periods and by implementing more stable genetic designs."*

We also added details about the timeline of the experiments in the result and the methods section, as well as the figure legends:

lines 211-213: "*… we focused on whether the engineered strain could establish in the honeybee gut, how long it could persist and whether it could sense arabinose in situ and provide spatial information on sugar availability*";

lines 225-226: "*Based on these observations, all subsequent measurements were performed 7 days post-colonization*";

lines 244-245: "*After 7 days, we dissected guts and imaged them by confocal microscopy*";

lines 663-665: "*… honeybee hindguts were collected seven days after inoculation with engineered S. alvi and Gilliamella, a time point selected to ensure sufficient biosensor abundance for reliable sugar detection, and …*";

Figure 3 legend; **Figure 4 legend**).

-The authors require additional controls to explicitly address how the presence of auto-fluorescent pollen granules or other autofluorescence in the gut may be impacting in vivo gfp measurements. This can be done by including a negative control in bee experiments colonized with wkB2 that does not express gfp in response to arabinose, and indicating the baseline gfp measurements in that case, in the presence and absence of pollen. For figure 4d, how can the authors verify extremely high GFP values are not the product of autofluorescent pollen granules? This could be addressed by performing an experiment like that reported in Figure 4 d-e with only the ac06 S. alvi.

Authors: We apologize for the lack of clarity in our original description, which may have understandably led to concerns regarding pollen autofluorescence. We are confident that our fluorescence measurements reflect bacterial GFP signal and are not significantly influenced by pollen-derived autofluorescence, for the following reasons:

(i) **Minimization of pollen presence prior to imaging.** In preliminary experiments, we observed that intact or partially degraded pollen grains could be present in the gut. To mitigate potential imaging issues, we amended our protocol and deprived bees used for imaging experiments of pollen for two days prior to dissection (as already described in our Materials and Methods, see **lines 645-646**). This substantially reduced the presence of pollen in the gut. In the rare instances where pollen grains were still present in the gut (*e.g.* Figures R1 below), these regions were excluded from imaging, and analysis was performed on adjacent regions devoid of visible pollen. We have now added this information in our methods, **lines 680-681**: "*Gut regions with visible pollen grains were excluded from imaging.*").

(ii) **Minimal autofluorescence of pollen in the green channel.** Importantly, pollen autofluorescence is predominantly observed in the red spectrum rather than in the green channel. In our experiments, this primarily posed a challenge for imaging *Gilliamella* cells labeled with E2-crimson, which overlap with pollen autofluorescence. This is the main reason we took care to avoid pollen-containing regions during imaging. We provided below for the reviewer two representative examples of gut sections imaged with pollen grains visible on the red channel (CRIM). As mentioned above, these type of images with visible pollen grains were not used to collect data shown in our manuscript but were taken as examples during test experiments carried out to optimize our protocols. The pollen grains, indicated for clarity by white arrows, emit minimal fluorescence in the green channel (GFP). Therefore, even if a small amount of residual pollen had inadvertently been included in the analyzed images, its minimal autofluorescence in the GFP channel would not be expected to significantly influence the measured GFP signal. We have now amended our results and methods to better reflect this distinction of pollen autofluorescence, **lines 293-295 and lines 644-646**: "*As pollen exhibits autofluorescence primarily in the red spectrum, which may interfere with E2-crimson signal detection, pollen was removed from cages two days prior to gut dissection*".

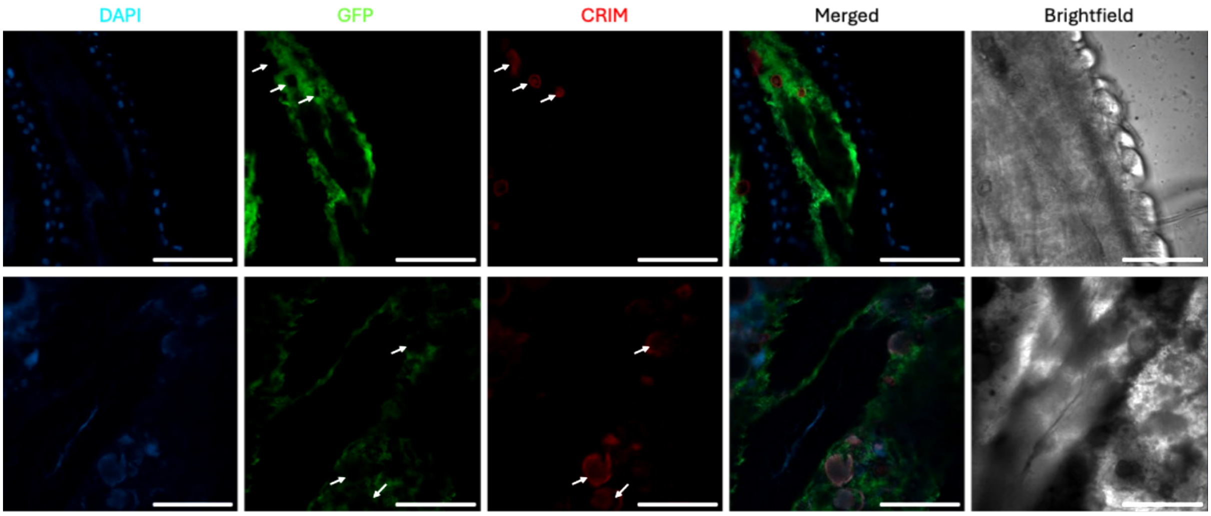

Figure R1: Examples of confocal microscopy images of a honeybee ileum colonized by engineered *S. alvi* ac02, with visible pollen grains. Two examples are shown (top and bottom rows). Bees were fed sugar water and pollen *ad libitum*. The blue channel depicts DAPI staining of host and bacterial DNA, the green channel shows GFP fluorescence from the engineered strain, the red channel shows autofluorescence of pollen grains. Scale bars represent 100 µm. Localizations of pollen grains are indicated by white arrows.

(iii) Segmentation and cell-specific signal quantification. Our image analysis pipeline relies on automated segmentation of bacterial cells based on a GFP fluorescence threshold identical across images (as already explained in our Methods **lines 683-691**). The resolution of our images mostly allows clear identification of individual bacterial cells, enabling precise segmentation. GFP fluorescence was quantified exclusively within these segmented bacterial regions, thereby preventing any contribution from potential pollen particles. We provide below for the reviewer two representative examples of gut sections analyzed using our pipeline. The first example (top row) shows segmentation on the same image shown Figure R1, with pollen grains, and the second example (bottom row) shows segmentation in case of co-colonization with *Gilliamella* cells. Our biosensor strain appears on the green channel (GFP), the *Gilliamella* cells appear on the red channel (CRIM). The 'cell segmentation' images indicate in a white mask the detected *S. alvi* bacterial regions based on the GFP signal.

Figure R2: Examples of confocal microscopy images of a honeybee ileum colonized by engineered *S. alvi* ac02, with visible pollen grains (top row) or co-colonized by engineered *S. alvi* ac02 and *G. apicola* ESL309 (bottom row). Bees were fed sugar water and pollen *ad libitum*. The blue channel depicts DAPI staining of host and bacterial DNA, the green channel shows GFP fluorescence from the engineered strain, the red channel shows autofluorescence of pollen grains and E2-crimson fluorescence from the *G. apicola* strain transformed with the pAC09 plasmid. Cell segmentation shows *S. alvi* bacterial regions, detected based on the GFP signal, as a white mask. Scale bar represents 100 µm.

(iv) **Assessment of background fluorescence.** Finally, to evaluate whether degraded pollen or non-visible pollen-derived particles could influence the measured GFP signal, we compared background fluorescence levels in guts from bees fed with and without pollen. We provide below for the reviewer an example analysis of two data points from Fig. 4d: one from the control condition (ESL169 / −) without pollen, and the highest fluorescence data point highlighted by the reviewer from the pollen condition (ESL169 / pollen). Both are indicated by black arrows and labelled A and B, respectively. Fluorescence intensity profiles were extracted along the indicated yellow lines on the corresponding microscopy images used to generate these data points, capturing both background regions and *S. alvi* biofilm. This analysis revealed no increase in background GFP signal in pollen-fed bees. Notably, if pollen-derived autofluorescent particles were contributing to the signal, they would be expected to be distributed throughout the gut lumen and thus elevate fluorescence broadly, including in background regions rather than being restricted to biofilm-associated areas. Accordingly, an increase in background fluorescence would be expected. However, this was not observed.

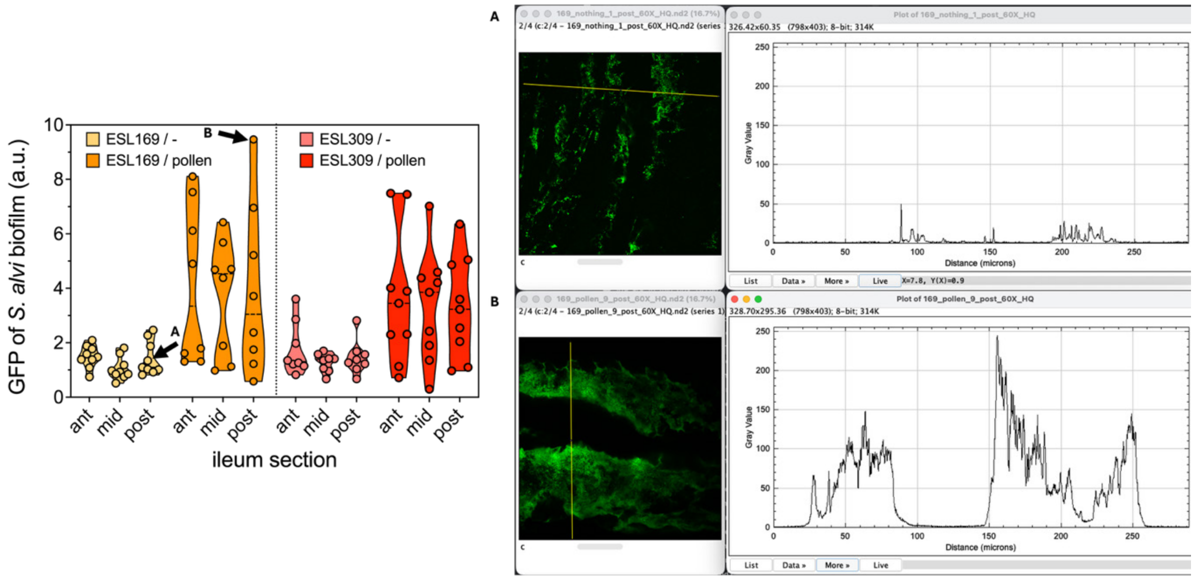

Figure R3: GFP fluorescence intensity profile from data points part of **Fig. 4d** indicated by arrows: (A) image representative without pollen and (B) with pollen. Yellow line indicates the region of interest analyzed (GFP channel, left). Line profile plot showing the raw pixel intensity corresponding to the transect (right).

Taken together, these controls provide strong evidence that the GFP measurements reported in our manuscript accurately reflect bacterial fluorescence rather than pollen autofluorescence.

-How do the authors justify the extremely high relative GFP in Fig 4e and Supp. Fig 8? Assuming the scales are comparable to earlier figures, this is much higher than bees fed 1mM arabinose (Fig 3e) with a "saturated" response. Effectively, these readings are outside the reported in vivo range of the biosensor. One possibility is that these high GFP values represent autofluorescent pollen granules, but possibly there are other interpretations. The authors should identify the biosensor dynamic range in vivo, in order to ensure the high GFP values are within range. Authors could repeat experiments like that in Fig 3c with increasing concentrations of arabinose to demonstrate the effective in vivo range of the biosensor, reaching levels approximate with the 10-15 a.u. shown in Fig 3c and Supp. Fig 8.

Authors: We thank the reviewer for raising this point and appreciate the opportunity to clarify.

First, the reviewer appears to interpret the response observed in Fig. 3g as reflecting saturation of the biosensor. However, our *in vitro* characterization demonstrates that the biosensor dynamic range extends up to ~6,700 µM arabinose (**Fig. 1e**), which is substantially higher than the concentrations used in our *in vivo* experiments (0, 10, 100, and 1,000 µM; Fig. 3c–g). Therefore, the response observed at 1,000 µM does not represent saturation of the biosensor itself. Furthermore, our GC-MS measurements indicate that the average arabinose levels derived from pollen in the gut remain within the detection range of the biosensor (see **lines 178-184** and **Fig. 2b**).

Importantly, the concentrations of arabinose provided to bees in sugar water do not directly correspond to the concentrations experienced by bacteria in the gut. Upon ingestion, the solution is subject to dilution, mixing, and spatial heterogeneity, resulting in local concentrations that are most likely both lower and unevenly distributed. The higher GFP values observed in Fig. 4e and Supplementary Fig. 9 (due to a change in figure numbers) are therefore most likely explained by locally elevated arabinose concentrations arising from pollen breakdown, that are simply higher than the ones resulting from the ingestion of a homogeneous arabinose solution, while not exceeding the biosensor's dynamic range (see again **Fig. 2b**). Consistent with this interpretation, we do not observe a plateau in GFP signal in these conditions, but rather high and distinct heterogenous values, further indicating that the sensor is operating within its responsive range.

Finally, we would like to emphasize that exploring substantially higher arabinose concentrations *in vivo* would not be physiologically relevant nor experimentally feasible, as concentrations above those tested in this study are known to be detrimental to bee viability, as discussed in our manuscript (see **lines 417-420** and reference Barker et al., "*Influence of diet on sugars found by thin-layer chromatography in thoraces of honey bees, Apis mellifera*", 1974, DOI: 10.1002/jez.1401880204). The concentrations examined here, which are within the range of biologically relevant arabinose levels as previously mentioned (**Fig. 2b**), were chosen to balance biological relevance with bee viability.

Taken together, these considerations support the idea that the high GFP values observed in Fig. 4e and Supplementary Fig. 9 fall within the operational range of the biosensor and do not reflect signal saturation or artefacts such as pollen autofluorescence.

-The most surprising findings are that there is no difference in arabinose throughout the bee gut in the presence of pollen (Fig 4d, e) despite a co-colonized gilliamella strain able to digest arabinose (Fig 4c). The authors suggest this is due to the high spatial resolution of their biosensor and micro-niches of high arabinose from pollen degradation throughout the gut that overwhelm gilliamella degradation. This is a reasonable interpretation, if the specificity and stability and dynamic range of the reporter are very robust. However, another interpretation is

that the biosensor readout is variable in the presence of pollen. The authors should confirm this overall finding using a bulk gc-ms measurement of arabinose (as in Fig. 2b) in bee guts colonized with S. alvi, G. apicola, and fed pollen. Correlating any in vivo measurement of arabinose using their biosensor with GC-MS on the same sample would greatly strengthen these results.

Authors: We thank the reviewer for this suggestion. In the answers to your previous points, we have provided additional data and clarifications demonstrating the specificity, stability, and dynamic range of our arabinose biosensor. We believe these address the concerns regarding potential variability of the biosensor readout and confirm that the strain reliably reports arabinose levels *in vivo*, including in the presence of pollen, supporting our current interpretation of the data.

Regarding the suggestion to use bulk GC-MS, while this method measures total arabinose (as in **Fig. 2b**), it cannot resolve the local micro-niche variations along the bee gut that are central to our findings shown in **Fig. 4d-e**. A key advantage of our biosensor is precisely its ability to capture such spatial heterogeneity, which is not accessible with bulk measurements. Consequently, correlating biosensor readouts with bulk GC-MS would not provide meaningful validation of the spatial patterns we report.

Thus, we believe that our current biosensor measurements are both robust and uniquely suited to capture the local arabinose dynamics described in this study.

Minor recommendations:

-Remove red squiggly underlines of random words throughout figures. They are meaningless and distracting

Authors: We have removed them.

-Numerous locations describing CFU abundance appear to be missing the "×" character- ie, lines 177 and 178 for example -In figure 1c the function of 4 engineered strain variants are reported. The specifics of these strains and their construction, however, are only found in the somewhat hard to decipher supplemental figure 3c. I recommend the authors either: (1) show only the dose response curve for ac02 in the main text and move ac3-6 data to the supplemental or (2) bring a streamlined table into the main figure that highlights the design differences between ac02-ac06 so that this chart is interpretable.

Authors: We have added the ‘x’ character throughout the manuscript and amended our Fig. 1 according to the reviewer’s suggestion. We now show only ac02 in the main text and moved ac03-06 to the supporting information.

-does the biosensor construct impact growth of S. alvi? The authors could characterize the in vitro growth of strain ac02 compared to wild-type wkB2 through growth curves or similar techniques. A significant growth defect impacts the genetic stability and spread of inactivating mutations. This is only a recommendation.

Authors: To determine whether the biosensor construct affects growth, we quantified CFU counts of *S. alvi* cells carrying the dual-plasmid system (pAC51b/c; +) or lacking the plasmids (-) following growth to stationary phase in the presence of different arabinose concentrations. These data are now included as **Supplementary Fig. 4c**. We detected no significant differences

in cell biomass either across arabinose concentrations or between plasmid-containing and plasmid-free strains, indicating that the biosensor system does not impose a measurable growth burden under the tested conditions.

We have added the conclusion of this experiment as part of the results, **lines 199-207** : "*To ensure that plasmid dynamics were not associated with a growth defect, we also assessed whether the biosensor constructs affect bacterial proliferation. We quantified CFU counts of* S. alvi *cells carrying the dual-plasmid system (pAC51b/c; +) or lacking the plasmids (-) following growth to stationary phase in the presence of different arabinose concentrations (Supplementary Fig. 4c). We detected no significant differences in cell biomass either across arabinose concentrations or between plasmid-containing and plasmid-free strains (two-way ANOVA, effect of plasmids: $F(1, 24) = 2.68$, p-value = 0.114; effect of arabinose: $F(5, 24) = 0.48$, p-value = 0.785), indicating that the biosensor system does not impose a measurable growth burden under the tested conditions*.".

-I am not quite sure how to interpret the in vivo GFP measurements reported (a.u.) in Figures 3c, e and similar. Are these values normalized by the DAPI present in the same cell or something else? Do a.u. values between 1-2 in figure 3e indicate only a 2-fold change despite a range of 0-1000 uM arabinose? This is much less sensitive than the fold change reported in 1c. Please include additional information about how these values are calculated and how they can be interpreted.

Authors: The reported GFP values (a.u.) correspond to GFP fluorescence normalized to the DAPI signal within the same segmented bacterial regions, as described in the Materials and Methods (see **lines 679–691**). This normalization accounts for differences in cell density and allows comparison across samples.

Indeed, the fold changes observed *in vivo* (*e.g.* Fig. 3e) are lower than those measured *in vitro* (Fig. 1e). We explain this by two main factors:

First, the arabinose concentrations experienced by bacteria *in vivo* are unlikely to match the nominal concentrations provided in the sugar solution. Upon ingestion, the solution is subject to dilution, mixing, and spatial heterogeneity along the gut, resulting in a narrower effective range of arabinose exposure than in controlled *in vitro* conditions.

Second, the *in vitro* measurements were obtained by flow cytometry at the single-cell level, providing highly sensitive quantification of the GFP signal. In contrast, *in vivo* measurements were carried out by confocal imaging of bacterial populations within the gut. Although our segmentation approach approximates individual cells, the GFP signal is quantified from optical sections, meaning that only a portion of each bacterial cell (rather than its full volume) might be captured. As a result, these measurements are inherently less sensitive and might have a reduced dynamic range compared to flow cytometry.
