# Supplementary material for: An engineered bacterial symbiont maps micron-scale sugar gradients in the honeybee gut
Source: PLoS Biol. 2026 Sep 16;24(9):e3003997. doi: 10.1371/journal.pbio.3003997 (PMC13608000; doi:10.1371/journal.pbio.3003997)
Supplement: Supplementary file 2 [file pbio.3003997.s019.docx]

**REVIEWS:**

Authors: We thank the reviewers for their thoughtful follow-up evaluation of our revised manuscript. We have carefully addressed all the remaining comments and provide a detailed point-by-point response below. We hope that the revisions satisfactorily address the reviewers' concerns and that the manuscript is now suitable for publication.

**Reviewer #1:**

The authors have addressed all my comments and concerns and have gone beyond what was expected. This is now a really great piece of work. Congratulations to all involved.

Authors: We thank the reviewer for their positive feedback on our revised manuscript and for their valuable insights throughout the review process.

----------------------------------------------------------------


**Reviewer #3:**

My minor comments have been addressed, but my major comments have not been addressed to a satisfactory extent. Further revisions are required, given it is an Update Article. I am not completely convinced.

1.      Surprisingly, many key reports on biosensors developed for potential applications in microbiota are missing, including, but not limited to, those from the laboratory of Pam Silver, Jeff Tabor, Tim Lu, Tae Seok Moon, Harris Wang, Matthew Chang et al. In addition, key reports on the bee gut microbiome are missing, including, but not limited to, that from the Jeffrey Barrick lab. The authors should perform extensive literature search to cite and discuss relevant previous work.

       The space/reference number limitation should not be the reason for extensive literature search and detailed discussion of prior work, which is the basis for every research project. Thus, I strongly suggest that they should conduct extensive literature searches to cite and discuss more relevant previous reports.

Authors: We have now expanded our introduction to discuss and cite previous reports of biosensors, including studies of all the laboratories mentioned by the reviewer (see **newly added** **references 29-40**).

**lines 63-70**: “*Indeed, engineered bacterial biosensors have emerged as a versatile platform for interrogating host-associated environments, enabling the detection of metabolites, inflammatory markers and other transient environmental cues^30–36^, as well as recording transient signals through genetic memory circuits^37,38^. These studies have demonstrated the feasibility of programmable microbial sensors* in vivo*, but their application to insect microbiomes has remained largely unexplored. In honeybees, recent advances in the genetic engineering of gut commensals have established the core symbiont* Snodgrassella alvi *as a chassis for microbiome engineering and functional studies^39–41^.”*

2.      I understand the challenges of developing biosensors in non-model organisms. However, the choice of sugar arabinose reduces my enthusiasm. I am less convinced regarding the utility and novelty of this work due to this choice and given that the authors developed another biosensor for IPTG in their previous report. Still, implementing this biosensor in the bee gut would be novel, but I would recommend a simple demonstration (not extensive though) of a third, more useful sensor in the bee gut because the proof-of-concept demonstration was made with the IPTG sensor.

       Demonstration of a third sensor would be beyond the scope of this work. In that case, the authors should include more extensive discussion of challenges and opportunities of developing sensors in such non-model organisms.

Authors: We have now expanded our discussion on challenges and opportunities of developing sensors in non-model organisms.

**lines 364-386**: “*Building on our previous work with synthetic inducers, the expanded toolkit now enables the implementation of more complex functionalities for nutrient biosensing within the gut environment, while providing a foundation for developing additional biosensors tailored to the diverse chemical signals encountered by symbionts during colonization.*

*Developing biosensors in non-model symbionts presents challenges beyond transferring an existing sensing module into a new host. In* S. alvi*, implementation of the arabinose sensor required functional uptake of the target molecule, development of genetic parts with appropriate expression levels, reliable chromosomal integration, and construction of a synthetic promoter that responds to arabinose in this organism. More generally, sensor performance in a native symbiont depends on the compatibility of its components with the bacterium’s genetic and physiological constraints, including endogenous regulatory and metabolic networks, genetic stability, and its physiological state during gut colonization. The genetic framework developed here addresses some of these challenges and provide a basis for future development, although each new sensing system will require specific optimization and validation to achieve robust in vivo performance, particularly when target molecules differ in their uptake or regulatory mechanisms.*

*A further challenge is matching sensor performance to the concentration range encountered in the native gut environment. Here, our arabinose biosensor can detect a biologically relevant range of arabinose concentrations with sufficient sensitivity. For applications requiring detection of lower concentrations, however, future iterations of the biosensor could increase sensitivity, for example, by incorporating a genetic amplification circuit^59^. At the same time, such enhancements would need to be balanced with maintaining stable and reliable performance* in vivo*.”*

**lines 454-458**: “*Expanding such approaches across hosts and microbial taxa will require continued development of genetic toolkits for undomesticated bacteria, an effort that remains a major bottleneck for applications beyond traditional laboratory organisms. As these capabilities mature, engineered symbionts will open new avenues to uncover the complex chemical dialogues that underpin host-microbe symbiosis.”*

----------------------------------------------------------------


**Reviewer #4:**

I appreciate the authors' thoughtful responses, generally agree with their arguments, and believe their additions overall strengthen their manuscript.

I have only one substantial remaining concern relating to the microscopy data and analysis presented in Figures 4d, 4e, and Supplemental Figures S8 and S9. The extremely high, and highly variable, "GFP of S. alvi" in some samples in the pollen conditions (~4-10 AU) remains surprising (Fig 4D-E). Previous experiments showed a max in vivo response of ~2 AU (1000 uM arabinose- Fig 2C-G), and ~3 AU (210 uM arabinose- Fig 4C). As the methods state, the "GFP of S. alvi biofilm" is the result of normalizing the GFP intensity channel to the DAPI intensity channel, with the expectation this controls for S. alvi cell density impacting the GFP signal. This is a somewhat reasonable, it not conventional, expectation but it can also introduce error if the DAPI signal itself is variable for some technical or batch reason different than the GFP. A lower-than-average DAPI signal in particular samples, even with a commensurate GFP signal, would lead to an elevated "normalized" signal. I
investigated a subset of "pollen" images "169_pollen_X_post_60X_HQ" the authors deposited into the BioImages database and found this to be the case. Several samples have a substantially lower overall DAPI signal than others, even for the eukaryotic nuclei, which should be comparable (see samples 4, 5, 7 vs 1, 2, 9). The non-pollen samples I looked at did not have this problem, with the DAPI signal overall consistent in the "ara" and "nothing" sets of 169. However, my review of the images was not comprehensive, and could be missing critical information the authors have about any batch processing or other factors. DAPI staining is notoriously variable and finicky, subject to penetrance, concentration, bleaching, and washing effects. It is often suitable to define cell locations, but not recommended to quantitatively correct for GFP.

I recommend the authors include the raw DAPI and GFP intensity values used to compute the "normalized" GFP value for all appropriate in vivo measurements as a supplemental table or supplemental figure in the manuscript. It is not clear to me that this DAPI normalization is necessary, but if the authors want to use it, they should additionally include the raw values. I attempted to use the provided FIJI macro to extract these values, but ran into technical challenges, though the macro appears sound.

Authors: We thank the reviewer for this careful evaluation of our microscopy analysis. We regret that we overlooked the potential influence of the pollen condition on DAPI fluorescence during our original analysis, and we appreciate the opportunity to correct this oversight. Following the reviewer's suggestion, we re-examined the raw microscopy data and found that the reviewer had indeed correctly identified a systematic reduction in DAPI fluorescence in pollen-fed bees.

To investigate this further, we segmented host cell nuclei using a consistent DAPI threshold across all images, quantified the mean DAPI fluorescence intensity per unit area, which we then compared with the DAPI signal measured in bacterial biofilms. As shown in the new **S11 Fig**, DAPI fluorescence was consistently lower in pollen-fed bees in both bacterial biofilms and host cell nuclei, with a similar proportional reduction. Importantly, all samples were stained simultaneously using the same DAPI solution and staining protocol, ruling out differences in staining batch as an explanation for the reduced DAPI fluorescence. This has now been clarified in our Methods section.

**lines 691-694**: “*A single master mix of sufficient volume for all samples was prepared and used for the entire staining procedure, ensuring that all samples were stained using the same DAPI solution and eliminating potential variation between staining batches”.*

Because host nuclei provide an internal cellular reference and all samples were stained simultaneously using the same DAPI solution and protocol, these results suggest that the reduced bacterial DAPI signal is unlikely to reflect differences in bacterial abundance, but rather a systematic reduction in DAPI fluorescence associated with the pollen condition. One possible explanation is that pollen-derived DNA or other pollen-associated components affect DAPI availability or staining efficiency.

Based on these observations, we calculated a correction factor (*k* = 0.6) from the relative DAPI fluorescence of host nuclei in pollen-fed *versus* non-pollen-fed bees (‘-‘ and ‘ara’ conditions) and used this factor to correct the DAPI measurements in pollen-fed samples prior to GFP normalization. Following this correction, the normalized GFP values are no longer inflated and are more consistent with the dynamic range observed in our other experiments (see revised **Fig. 4d, Fig. 4e, S8 Fig and S9 Fig**).

The Methods section has been updated accordingly to describe the revised normalization procedure.

**lines 711-725**: “*Within each GFP-segmented region, mean fluorescence intensity per unit area was then extracted for both GFP and DAPI channels. For host cell nuclei, regions of interest were instead segmented by applying an identical DAPI fluorescence threshold across images. The threshold was selected to preferentially identify the high-intensity DAPI signal from host cell nuclei while excluding the majority of bacterial DAPI signal. Mean DAPI fluorescence intensity per unit area was then quantified within each DAPI-segmented region. As DAPI fluorescence was reduced to a similar extent in both bacterial biofilm and host cell nuclei in pollen-fed bees, despite identical staining conditions, a DAPI normalization factor (*k *= 0.6) was calculated as the ratio of mean DAPI fluorescence in pollen-fed bees to that in the other diet conditions (****S11 Fig****). DAPI values from pollen-fed bees were then divided by* k *to obtain corrected DAPI values. The observed reduction in DAPI signal in pollen-fed bees may reflect effects of pollen-derived DNA or other pollen-associated material on DAPI availability or staining efficiency. GFP intensities were then normalized to the corrected DAPI intensities measured from the same segmented area to account for variation in cell density^67-68^.”*

In response to the reviewer's recommendation, we have also included the raw GFP and DAPI fluorescence measurements underlying these analyses in the source file **S1 Data file**.

Regarding the reviewer’s comment that normalization by DAPI is unconventional, we would like to mention that this approach has been reported in the literature, for example, <https://doi.org/10.1021/acssynbio.5c00193> and in <https://doi.org/10.1007/s00253-020-11051-7>. We have now added those references to the methods:

**lines 724-725**: “*GFP intensities were then normalized to the corrected DAPI intensities measured from the same segmented area to account for variation in cell density^67-68^.”*

**EDITORIAL REQUESTS:**

1) We routinely suggest changes to titles to ensure maximum accessibility for a broad, non-specialist readership, and to ensure they reflect the contents of the paper. In this case, we would suggest a minor edit to the title, as follows. Please ensure you change both the manuscript file and the online submission system, as they need to match for final acceptance:

"An engineered bacterial symbiont maps micron-scale sugar gradients in the honeybee gut"

Authors: We title has been modified.

2) Please add the weblink of the funding agencies in the Financial Disclosure statement in the manuscript details.

Authors: The weblinks of the funding agencies were added.

3) I have noticed that in response to reviewers you mentioned some data that is not in the manuscript, like the concentrations of other pentose sugars. We would like to encourage to add the data to the manuscript or to opt for open peer-review so the readers can also have access to this information.

Authors: We are happy to opt for open peer-review.

4) Thank you for providing the raw values of the data underlying the figures. Please cite the location of the data clearly in all relevant main and supplementary Figure legends, e.g. “The data underlying this Figure can be found in S1 Data” or “The data underlying this Figure can be found in [https://doi.org/10.5281/zenodo.XXXXX”](https://doi.org/10.5281/zenodo.XXXXX%E2%80%9D) 

Authors: The location of the data is now clearly cited.

5) For figures containing FACS data, please provide the FCS files. We ask that you please deposit this data in an open repository like Zenodo, and provide the accession number/URL of the deposition in the Data Availability Statement in the online submission form.

Authors: We have uploaded the FCS files for Fig. 2a and S5 Fig. to Zenodo (DOI: 10.5281/zenodo.22029676). However, regarding figures Fig. 1, Fig. 1e, S2b Fig. and S10 Fig., the original FCS files underlying the FACS data presented in this manuscript were permanently deleted after the first author departed the laboratory and are no longer available. We sincerely regret this limitation and apologize for any inconvenience caused.

Please note that we were unaware of a requirement to submit raw FCS files. In our previous PLoS Biology publication this was not requested. Nevertheless, we believe that we align with your policy, which states: “*Also, authors do not need to submit the raw data collected during an investigation if the standard in the field is to share data that have been processed*”. Indeed, the deposition of raw FCS files is not standard practice in synthetic biology.

As an alternative, we have retained the original PDF analysis reports, which include the analytical plots and the corresponding numerical value tables generated from the FCS files prior to their deletion. These reports are available from the corresponding author upon request. Additionally, the gating strategy is detailed in S10 Fig.

6) Please ensure that your Data Statement in the submission system accurately describes where your data can be found and is in final format, as it will be published as written there.

Authors: The Data Statement has been reviewed and is in its final form.

7) Per journal policy, if you have generated any custom code during the course of this investigation, please make it available without restrictions. I am aware that you have provide some code as S1 Text in an image, but this is not easily accessible. Please ensure that the code is sufficiently well documented and reusable, and that your Data Statement in the Editorial Manager submission system accurately describes where your code can be found. More information on our Code Policy, what and how to share can be found here: <https://journals.plos.org/plosbiology/s/code-availability>

Please note that we cannot accept sole deposition of code in GitHub, as this could be changed after publication. However, you can archive this version of your publicly available GitHub code to Zenodo. Once you do this, it will generate a DOI number, which you will need to provide in the Data Accessibility Statement (you are welcome to also provide the GitHub access information). See the process for doing this here: <https://docs.github.com/en/repositories/archiving-a-github-repository/referencing-and-citing-content>

Authors: The FIJI code was made available to Zenodo and its corresponding DOI number has been added to the Data Statement.

In addition to these revisions, you may need to complete some formatting changes, which you will receive in a follow up email. A member of our team will be in touch with a set of requests shortly. If you do not receive a separate email within a few days, please assume that checks have been completed, and no additional changes are required.

Authors: We have addressed all formatting requests.
