# Supplementary material for: An engineered bacterial symbiont maps micron-scale sugar gradients in the honeybee gut
Source: PLoS Biol. 2026 Sep 16;24(9):e3003997. doi: 10.1371/journal.pbio.3003997 (PMC13608000; doi:10.1371/journal.pbio.3003997)
Supplement: Peer review history

# Editor report: Decision Letter 0

**DOI:** 10.1371/journal.pbio.3003997.r001

**Authors:** Richard Hodge

5 Feb 2026

Dear Yolanda,

Thank you for submitting your manuscript entitled "Engineered symbiont biosensor maps micron-scale sugar gradients in the honeybee gut" for consideration as a Methods and Resources Article by PLOS Biology.

Your manuscript has now been evaluated by the PLOS Biology editorial staff, as well as by an academic editor with relevant expertise, and I am writing to let you know that we would like to send your submission out for external peer review.

IMPORTANT: After discussions with the rest of the editorial team, we think your manuscript would be a good fit in our ‘Update Article’ format (https://journals.plos.org/plosbiology/s/what-we-publish#loc-update-article). This is in relation to your previously published study in PLOS Biology (Ref 30), since it expands on the initial feasibility study and now represents the first bacterial biosensor engineered to sense a biologically relevant metabolite in the bee gut. During resubmission (see guidelines below), I would be grateful if you could please tick ‘Update Article’ as the article type in the dropdown menu of the online submission form.

Before we can send your manuscript to reviewers, we need you to complete your submission by providing the metadata that is required for full assessment. To this end, please login to Editorial Manager where you will find the paper in the 'Submissions Needing Revisions' folder on your homepage. Please click 'Revise Submission' from the Action Links and complete all additional questions in the submission questionnaire.

Once your full submission is complete, your paper will undergo a series of checks in preparation for peer review. After your manuscript has passed the checks it will be sent out for review. To provide the metadata for your submission, please Login to Editorial Manager (https://www.editorialmanager.com/pbiology) within two working days, i.e. by Feb 07 2026 11:59PM.

If your manuscript has been previously peer-reviewed at another journal, PLOS Biology is willing to work with those reviews in order to avoid re-starting the process. Submission of the previous reviews is entirely optional and our ability to use them effectively will depend on the willingness of the previous journal to confirm the content of the reports and share the reviewer identities. Please note that we reserve the right to invite additional reviewers if we consider that additional/independent reviewers are needed, although we aim to avoid this as far as possible. In our experience, working with previous reviews does save time.

If you would like us to consider previous reviewer reports, please edit your cover letter to let us know and include the name of the journal where the work was previously considered and the manuscript ID it was given. In addition, please upload a response to the reviews as a 'Prior Peer Review' file type, which should include the reports in full and a point-by-point reply detailing how you have or plan to address the reviewers' concerns.

During the process of completing your manuscript submission, you will be invited to opt-in to posting your pre-review manuscript as a bioRxiv preprint. Visit http://journals.plos.org/plosbiology/s/preprints for full details. If you consent to posting your current manuscript as a preprint, please upload a single Preprint PDF.

Feel free to email us at plosbiology@plos.org if you have any queries relating to your submission.

Kind regards,

Richard

Richard Hodge, PhD

Senior Editor, PLOS Biology

rhodge@plos.org

PLOS

Empowering researchers to transform science

Carlyle House, Carlyle Road, Cambridge, CB4 3DN, United Kingdom

California (U.S.) corporation #C2354500, based in San Francisco

# Peer review: Decision Letter 1

**DOI:** 10.1371/journal.pbio.3003997.r002

**Authors:** Richard Hodge

25 Mar 2026

Dear Yolanda,

Thank you for your patience while your manuscript "Engineered symbiont biosensor maps micron-scale sugar gradients in the honeybee gut" was peer-reviewed at PLOS Biology as an Update Article. Please accept my sincere apologies for the delays that you have experienced during the peer review process. Your manuscript has now been evaluated by the PLOS Biology editors, an Academic Editor with relevant expertise, and by four independent reviewers.

In light of the reviews, which you will find at the end of this email, we would like to invite you to revise the work to thoroughly address the reviewers' reports.

As you can see, the reviewers are generally positive about your manuscript and think it is well done and provides an interesting update to the original report. Reviewer #1 is supportive of publication and asks that several improvements to the text and figure presentation are provided. Reviewer #2 raises concerns with the characterization of plasmid stability and asks that later time points are analysed. In addition, Reviewer #3 raises some general concerns about the choice of arabinose as the model metabolite, recommending that a simple demonstration of a third sensor in the bee gut is provided, and that further contextualization and reference of the previous literature is included. Finally, Reviewer #4 provides the most extensive experimental requests and raises concerns with the overall characterization of the biosensor, including its arabinose specificity, genetic stability and demonstration of high dynamic range.

Given the extent of revision needed, we cannot make a decision about publication until we have seen the revised manuscript and your response to the reviewers' comments. Your revised manuscript is likely to be sent for further evaluation by all or a subset of the reviewers.

In addition to these revisions, you will need to complete some formatting changes, which you will receive in a follow up email. A member of our team will be in touch with a set of requests shortly.

We expect to receive your revised manuscript within 3 months. Please email us (plosbiology@plos.org) if you have any questions or concerns, or would like to request an extension.

At this stage, your manuscript remains formally under active consideration at our journal; please notify us by email if you do not intend to submit a revision so that we may withdraw it.

**IMPORTANT - SUBMITTING YOUR REVISION**

Your revisions should address the specific points made by each reviewer. Please submit the following files along with your revised manuscript:

1. A 'Response to Reviewers' file - this should detail your responses to the editorial requests, present a point-by-point response to all of the reviewers' comments, and indicate the changes made to the manuscript.

*NOTE: In your point-by-point response to the reviewers, please provide the full context of each review. Do not selectively quote paragraphs or sentences to reply to. The entire set of reviewer comments should be present in full and each specific point should be responded to individually, point by point.

You should also cite any additional relevant literature that has been published since the original submission and mention any additional citations in your response.

2. In addition to a clean copy of the manuscript, please also upload a 'track-changes' version of your manuscript that specifies the edits made. This should be uploaded as a "Revised Article with Changes Highlighted" file type.

*Re-submission Checklist*

When you are ready to resubmit your revised manuscript, please refer to this re-submission checklist: https://plos.io/Biology_Checklist

To submit a revised version of your manuscript, please go to https://www.editorialmanager.com/pbiology/ and log in as an Author. Click the link labelled 'Submissions Needing Revision' where you will find your submission record.

Please make sure to read the following important policies and guidelines while preparing your revision:

*Published Peer Review*

Please note while forming your response, if your article is accepted, you may have the opportunity to make the peer review history publicly available. The record will include editor decision letters (with reviews) and your responses to reviewer comments. If eligible, we will contact you to opt in or out. Please see here for more details:

https://blogs.plos.org/plos/2019/05/plos-journals-now-open-for-published-peer-review/

*PLOS Data Policy*

Please note that as a condition of publication PLOS' data policy (http://journals.plos.org/plosbiology/s/data-availability) requires that you make available all data used to draw the conclusions arrived at in your manuscript. If you have not already done so, you must include any data used in your manuscript either in appropriate repositories, within the body of the manuscript, or as supporting information (N.B. this includes any numerical values that were used to generate graphs, histograms etc.). For an example see here: http://www.plosbiology.org/article/info%3Adoi%2F10.1371%2Fjournal.pbio.1001908#s5

*Blot and Gel Data Policy*

We require the original, uncropped and minimally adjusted images supporting all blot and gel results reported in an article's figures or Supporting Information files. We will require these files before a manuscript can be accepted so please prepare them now, if you have not already uploaded them. Please carefully read our guidelines for how to prepare and upload this data: https://journals.plos.org/plosbiology/s/figures#loc-blot-and-gel-reporting-requirements

*Protocols deposition*

To enhance the reproducibility of your results, we recommend that if applicable you deposit your laboratory protocols in protocols.io, where a protocol can be assigned its own identifier (DOI) such that it can be cited independently in the future. Additionally, PLOS ONE offers an option for publishing peer-reviewed Lab Protocol articles, which describe protocols hosted on protocols.io. Read more information on sharing protocols at https://plos.org/protocols?utm_medium=editorial-email&utm_source=authorletters&utm_campaign=protocols

Thank you again for your submission to our journal. We hope that our editorial process has been constructive thus far, and we welcome your feedback at any time. Please don't hesitate to contact us if you have any questions or comments.

Best regards,

Richard

Richard Hodge, PhD

Senior Editor, PLOS Biology

rhodge@plos.org

------------------------------------

REVIEWS:

Reviewer #1: In this study, Chhun et al. present a biosensor designed to provide insight into spatial gradients of arabinose metabolism. Using newly characterised promoters, the authors demonstrate the successful domestication of a non-native arabinose-inducible circuit for functionality in Snodgrassella alvi. The dynamic range of the system was fine-tuned by modulating expression levels of the genes araE and araC through newly screened promoters, as well as by altering plasmid backbones to control the copy number of either the araC transcription factor or the regulated GFP reporter. Building on this design, the authors demonstrate that the biosensor circuit remains stable even in the absence of selection and exhibits strong specificity, with induction occurring exclusively in response to arabinose. Finally, they showcase the utility of the biosensor in resolving both longitudinal and transversal spatial variation in arabinose concentrations. This is achieved by deploying the S. alvi biosensor in mono-colonised bees or in co-colonisation with different Gilliamella species, under conditions where bees were fed either pure arabinose or pollen-derived arabinose.

This work represents a significant advance over existing inducible genetic circuits in S. alvi, which have largely relied on the non-physiologically relevant inducer IPTG. By contrast, this study demonstrates that a biosensor can be used to monitor biologically relevant sugar metabolites in a spatially resolved manner. Overall, the study highlights the power of in vivo gut biosensors and is likely to inspire future efforts to engineer additional biosensors in other honeybee gut symbionts, enabling deeper insight into key metabolic processes within the honeybee gut microbiome and their broader implications for honeybee holobiont physiology. This should be of interest to the readership of PLoS Biology who will appreciate the coming together of microbiology, molecular biology, insect biology, bioengineering and environmental research that this study represents. It is high quality interdisciplinary work that should interest a broad readership.

Below are a list of minor comments which need to be addressed:

1.In numerous figures, some of the text has a red error line which normally indicates a spelling error. This is seen in Fig 1a - "sensing", Fig 3b - "Merged" and "Brightfield", Fig 4a - "biosensor", "S. alvi" and "days", "G. apicola, "Colonization", "Cup-housed", "gut", "analysis" and "days", Fig 4b - "S. alvi and "G. apicola". Please correct this in the figures.

2.The dose response curve in Fig 1c shows the fold-change activity at the lowest non-inducing concentration relative to the uninduced conditions across the various designs is 1.4-fold. It would be wise to comment on, perhaps in the discussion, as to how this signal could be boosted to improve the signal to noise ratio further (maybe through amplification circuits?). In addition, please also refer to Armbruster and Pry (2008) (https://pmc.ncbi.nlm.nih.gov/articles/PMC2556583/) to determine the true detection limit of the S. alvi biosensor which needs to be reported, especially given that the smaller reported induction values of 1.1-1.4-fold may just be within the analytical noise of the biosensor.

3.In all diagrams which depict genetic circuits, the promoters have been annotated, but other key modular parts such as RBSs or terminators have not been annotated. These should be corrected.

4.In Supplementary Fig. 3c, the images depicting an overview of the biosensor strains are quite pixelated. This should be replaced with a higher quality version.

5.The authors present newly characterized promoters for S. alvi in Supplementary Fig. 1. I suggest incorporating this figure as an additional subpanel within Fig. 1. Doing so would better justify the selection of specific promoters used for circuit design and make these genetic parts more visible and accessible to readers interested in applying them in future work. There appears to be sufficient space in Fig. 1 to accommodate this addition. In addition, Supplementary Fig. 3a-b could be integrated as further subpanels in Fig. 1 to more clearly illustrate the engineering strategy used to generate a functional arabinose-inducible promoter. Specifically, it would be valuable to include colony images for all pBAD designs, rather than only the single uninduced and induced examples currently shown in Fig. 1b. The domestication of inducible promoters for use in non-model organisms is technically challenging, and the substantial effort undertaken here to establish a functional system in S. alvi merits more prominent presentation in the main figures. The text accompanying Fig. 1 should be revised accordingly to reflect these additions and to explicitly highlight the rationale for promoter selection as well as the iterative design process underlying the development of the inducible system.

6.Lines 166 to 169: "While part of the biosensor design was chromosomally integrated, we hypothesized that plasmid-borne elements might be lost under non-selective conditions, as expected in the natural gut environment". If stable genome integration is achievable for parts of the biosensing circuit, such as the araE gene which is integrated at the tetC locus shown in this work, could other components of the circuit also not have been genome integrated to enhance the stability of the circuits (i.e the araC gene and the reporter construct) - either at the same locus at araE or at different loci? Please comment.

7.Lines 160-165: "Using this normalization for the gut volume, arabinose concentrations averaged 2.2 ± 1.1 mM in MD bee guts, and 0.0722 ± 0.0164 mM in CB bee guts (n = 8; Fig. 2b). This range falls within the detection window of the engineered S. alvi strain ac02 (Fig. 1c), suggesting it can report variations in arabinose concentrations of both extremes: high concentrations in the absence of microbial metabolism (i.e. MD bees) and reduced, ecologically relevant levels when the native gut community consumes arabinose (i.e. CB bees)." � In this section, can the claim "ecologically relevant levels when the native gut community consumes arabinose" be true? This experimental set up does not comprise a complete honeybee native gut microbiome community and therefore technically not ecologically relevant in the context of the experiment conducted. In which case, the sentence should be revised to reflect this.

8.As a comment which follows on from point 7, the authors mention "previous studies have identified Gilliamella and Bifidobacterium as the principal degraders of complex carbohydrates in the bee gut" - lines 60 to 61. How different would the experimentally determined arabinose concentrations (0.0722 mM) be in the presence of other microbiome members which also metabolise complex carbohydrates such as arabinose? If more microbiome members are metabolising carbohydrates, I would suspect that the levels may end up below the detection range of the biosensor. Would this not impact the utility of an arabinose biosensor under a complete microbiome community? It would be wise to make a comment on this in the text.

9.The authors report values of "2.2 ± 1.1 mM in MD bee guts, and 0.0722 ± 0.0164 mM in CB bee guts", please convert these to µM values so that it makes it easier to infer where they are on dose response curve of biosensor S. alvi ac02 (Fig. 1c) where the x-axis units are µM.

10.Figure 2c investigates the stability of the genetic circuit in liquid culture in the absence and presence of antibiotic selection. As the paper focuses on the utility of a biosensor for in vivo monitoring, is this figure relevant in the context of the rest of the paper considering it is an experiment not representative of the gut environment?

11.In relation to Fig 3a which demonstrates the bacterial load in the absence and presence of antibiotic selection, could a similar style of experiment not have been performed to assess plasmid stability at day 7 and 14 in vivo?

12.Figure 3b caption: "A confocal microscopy image of a honeybee gut colonized by engineered S. alvi ac02 in the ileum region. A bacterial biofilm within a gut crypt is shown. The blue channel depicts DAPI staining of host and bacterial DNA and the green channel shows GFP fluorescence from the engineered strain". Whie in the main body of the text it is mentioned that Fig 3b depicts basal GFP levels in the absence of induction, please make it clear in the caption that this is in the absence of arabinose.

Reviewer #2: The authors engineer S. alvi as a living biosensor for arabinose in the honeybee gut, achieving micron-scale spatial resolution of sugar gradients within bacterial biofilms. The paper advances the field on two fronts: expanding the genetic toolkit for a non-model symbiont and applying it to reveal previously inaccessible nutrient microenvironments in vivo. The work is well-suited for PLOS Biology. However, there is a minor issue that should be addressed.

Plasmid stability. Supplementary Figure 5a indicates that the araC plasmid does not achieve 100% maintenance even under antibiotic selection. What might explain this incomplete retention?

In addition, plasmid stability was assessed only after 3 days in vitro (Fig. 2c), whereas in vivo imaging was performed at day 7. Plasmid maintenance at this later time point, particularly in the absence of antibiotic selection, could be substantially lower. Since Figure 3a already involves selective plating of fecal samples, performing parallel plating on single-antibiotic and no-antibiotic plates would enable calculation of in vivo plasmid retention at day 7, which would strengthen the analysis.

Reviewer #3: This exciting and solid work demonstrates a simple biosensor and its utility in the honeybee gut. While relatively well-designed, this work would be stronger if the following points were addressed.

1.Surprisingly, many key reports on biosensors developed for potential applications in microbiota are missing, including, but not limited to, those from the laboratory of Pam Silver, Jeff Tabor, Tim Lu, Tae Seok Moon, Harris Wang, Matthew Chang et al. In addition, key reports on the bee gut microbiome are missing, including, but not limited to, that from the Jeffrey Barrick lab. The authors should perform extensive literature search to cite and discuss relevant previous work.

2.I understand the challenges of developing biosensors in non-model organisms. However, the choice of sugar arabinose reduces my enthusiasm. I am less convinced regarding the utility and novelty of this work due to this choice and given that the authors developed another biosensor for IPTG in their previous report. Still, implementing this biosensor in the bee gut would be novel, but I would recommend a simple demonstration (not extensive though) of a third, more useful sensor in the bee gut because the proof-of-concept demonstration was made with the IPTG sensor.

3.When comparing data, I suggest that authors should use statistical analysis with p-value reported in the figure caption and figures if possible.

4.Given that biosensors have been developed for microbiome applications for more than a decade, the authors need to tone down, minimizing overclaiming.

5.Readers would appreciate it if the authors discuss the limitations of their approach using this sensor in more details.

Reviewer #4: In this manuscript the authors create an arabinose "in vivo biosensor" strain of S. alvi, expanding their previous work demonstrating IPTG induction in this bee gut associated bacterium. They demonstrate chromosomal integration of the araE transporter as well as a two-plasmid sense and respond circuit that expresses GFP in response to the presence of arabinose in a dose dependent manner. The authors then test this circuit's function in the context of the bee gut and the presence or absence of pollen and co-colonizing G. apicola strains. The authors report on potential arabinose gradients in the bee gut and identify highly variable arabinose-rich micro-environments as the result of pollen degradation. They propose the biosensor as a highly sensitive and spatially resolved reporter of arabinose concentration in vivo.

Overall, this work is an appropriate and interesting update to the initial report. I welcome the expanded engineering approaches in bee gut bacteria and the use of engineered microbes as sensitive biosensors for complex and inaccessible gut environs. I have several significant concerns, however, which may undermine the reported findings unless they are addressed. To reliably trust the biosensor output the authors should strongly demonstrate the sensor is specific to arabinose, is genetically stable, and has a high dynamic range both in vitro and in vivo.

Major concerns:

-The characterization of the biosensor's arabinose specificity is insufficient. As the authors correctly point out, the bee gut is a complex environment rich in carbohydrates. Testing alternative sugars at a single concentration as in Figure 2a is a good first step. However, sugar concentration impacts sensor function and araE can import alternative sugars, possibly impacting S. alvi physiology. To verify specificity, the authors should further characterize the biosensor in the laboratory across a range of concentrations of ribose and xylose, and test 50:50 mixtures of arabinose with alternative sugars. Ideally, the authors would also demonstrate that the biosensor is not activated in bees colonized with ac02 and fed ribose or xylose. However, this experiment is substantial work and this caveat about specificity in vivo could instead be addressed in the discussion.

-I have concerns about the genetic stability of the biosensor construct and how that may impact the sensor's function in the bee gut. A biosensor construct that is too burdensome or prone to mutation is not a reliable reporter. Testing plasmid maintenance in vitro is an important but incomplete method to assess stability. For example, strains that accumulate non-functional mutations but maintain the plasmid are not captured in this assay but can be a substantial portion of cells, especially when constructs are burdensome. The fact that ~50% of colonies lose a plasmid in a single culture in the absence of antibiotic selection suggest substantial burden. The authors should repeat this stability assay and select colonies that maintain both plasmids and then test their function in the presence and absence of arabinose. As above, the strongest evidence would see the same stability demonstrated in bacterial colonies isolated after some period of colonization in honey bees from an experiment like that shown in Fig 3a. However, this could instead be addressed as a possible source of error in the discussion.

-The authors require additional controls to explicitly address how the presence of auto-fluorescent pollen granules or other autofluorescence in the gut may be impacting in vivo gfp measurements. This can be done by including a negative control in bee experiments colonized with wkB2 that does not express gfp in response to arabinose, and indicating the baseline gfp measurements in that case, in the presence and absence of pollen. For figure 4d, how can the authors verify extremely high GFP values are not the product of autofluorescent pollen granules? This could be addressed by performing an experiment like that reported in Figure 4 d-e with only the ac06 S. alvi.

-How do the authors justify the extremely high relative GFP in Fig 4e and Supp. Fig 8? Assuming the scales are comparable to earlier figures, this is much higher than bees fed 1mM arabinose (Fig 3e) with a "saturated" response. Effectively, these readings are outside the reported in vivo range of the biosensor. One possibility is that these high GFP values represent autofluorescent pollen granules, but possibly there are other interpretations. The authors should identify the biosensor dynamic range in vivo, in order to ensure the high GFP values are within range. Authors could repeat experiments like that in Fig 3c with increasing concentrations of arabinose to demonstrate the effective in vivo range of the biosensor, reaching levels approximate with the 10-15 a.u. shown in Fig 3c and Supp. Fig 8.

-The most surprising findings are that there is no difference in arabinose throughout the bee gut in the presence of pollen (Fig 4d, e) despite a co-colonized gilliamella strain able to digest arabinose (Fig 4c). The authors suggest this is due to the high spatial resolution of their biosensor and micro-niches of high arabinose from pollen degradation throughout the gut that overwhelm gilliamella degradation. This is a reasonable interpretation, if the specificity and stability and dynamic range of the reporter are very robust. However, another interpretation is that the biosensor readout is variable in the presence of pollen. The authors should confirm this overall finding using a bulk gc-ms measurement of arabinose (as in Fig. 2b) in bee guts colonized with S. alvi, G. apicola, and fed pollen. Correlating any in vivo measurement of arabinose using their biosensor with GC-MS on the same sample would greatly strengthen these results.

Minor recommendations:

-Remove red squiggly underlines of random words throughout figures. They are meaningless and distracting

-Numerous locations describing CFU abundance appear to be missing the "×" character- ie, lines 177 and 178 for example

-In figure 1c the function of 4 engineered strain variants are reported. The specifics of these strains and their construction, however, are only found in the somewhat hard to decipher supplemental figure 3c. I recommend the authors either: (1) show only the dose response curve for ac02 in the main text and move ac3-6 data to the supplemental or (2) bring a streamlined table into the main figure that highlights the design differences between ac02-ac06 so that this chart is interpretable.

-does the biosensor construct impact growth of S. alvi? The authors could characterize the in vitro growth of strain ac02 compared to wild-type wkB2 through growth curves or similar techniques. A significant growth defect impacts the genetic stability and spread of inactivating mutations. This is only a recommendation.

-I am not quite sure how to interpret the in vivo GFP measurements reported (a.u.) in Figures 3c, e and similar. Are these values normalized by the DAPI present in the same cell or something else? Do a.u. values between 1-2 in figure 3e indicate only a 2-fold change despite a range of 0-1000 uM arabinose? This is much less sensitive than the fold change reported in 1c. Please include additional information about how these values are calculated and how they can be interpreted.

# Author comment: Author response to Decision Letter 2

**DOI:** 10.1371/journal.pbio.3003997.r003

6 Jul 2026

Attachment Submitted filename: Rebuttal.pdf

# Peer review: Decision Letter 2

**DOI:** 10.1371/journal.pbio.3003997.r004

**Authors:** Richard Hodge

31 Jul 2026

Dear Dr Schaerli,

Thank you for your patience while we considered your revised manuscript "Engineered symbiont biosensor maps micron-scale sugar gradients in the honeybee gut" for consideration as a Update Article at PLOS Biology. Your revised study has now been evaluated by the PLOS Biology editors, the Academic Editor and three of the original reviewers.

As you will see in the reports, all reviewers think recognize the good job done addressing their previous concerns, but Reviewers #3 and #4 still would like some aspects to be addressed. Reviewer 3 says that the manuscript still omits relevant literature on microbiota biosensors and the bee gut microbiome, and that the use of arabinose limits the perceived novelty and utility of the work. The reviewer suggests either demonstrating a third, more practically relevant sensor in the bee gut or, if this is beyond scope, substantially expanding the discussion of the challenges and opportunities associated with sensor development in non-model organisms. Reviewer 4 still raises a concern about the microscopy analysis in Figures 4d–e and S8–S9, saying that variability in DAPI staining may artifactually inflate normalized GFP values in some pollen samples, and recommends providing the raw DAPI and GFP intensity measurements for all relevant in vivo experiments in a supplementary table or figure.

IMPORTANT: While the experimental requests from reviewer #3 will strengthen the conclusion of the study, we think this is out of scope and a discussion of the limitations, challenges and opportunities would suffice.

Additionally, please address the following editorial requests:

1) We routinely suggest changes to titles to ensure maximum accessibility for a broad, non-specialist readership, and to ensure they reflect the contents of the paper. In this case, we would suggest a minor edit to the title, as follows. Please ensure you change both the manuscript file and the online submission system, as they need to match for final acceptance:

"An engineered bacterial symbiont maps micron-scale sugar gradients in the honeybee gut"

2) Please add the weblink of the funding agencies in the Financial Disclosure statement in the manuscript details.

3) I have noticed that in response to reviewers you mentioned some data that is not in the manuscript, like the concentrations of other pentose sugars. We would like to encourage to add the data to the manuscript or to opt for open peer-review so the readers can also have access to this information.

4) Thank you for providing the raw values of the data underlying the figures. Please cite the location of the data clearly in all relevant main and supplementary Figure legends, e.g. “The data underlying this Figure can be found in S1 Data” or “The data underlying this Figure can be found in https://doi.org/10.5281/zenodo.XXXXX”

5) For figures containing FACS data, please provide the FCS files. We ask that you please deposit this data in an open repository like Zenodo, and provide the accession number/URL of the deposition in the Data Availability Statement in the online submission form.

6) Please ensure that your Data Statement in the submission system accurately describes where your data can be found and is in final format, as it will be published as written there.

7) Per journal policy, if you have generated any custom code during the course of this investigation, please make it available without restrictions. I am aware that you have provide some code as S1 Text in an image, but this is not easily acceisble. Please ensure that the code is sufficiently well documented and reusable, and that your Data Statement in the Editorial Manager submission system accurately describes where your code can be found. More information on our Code Policy, what and how to share can be found here: https://journals.plos.org/plosbiology/s/code-availability

Please note that we cannot accept sole deposition of code in GitHub, as this could be changed after publication. However, you can archive this version of your publicly available GitHub code to Zenodo. Once you do this, it will generate a DOI number, which you will need to provide in the Data Accessibility Statement (you are welcome to also provide the GitHub access information). See the process for doing this here: https://docs.github.com/en/repositories/archiving-a-github-repository/referencing-and-citing-content

In addition to these revisions, you may need to complete some formatting changes, which you will receive in a follow up email. A member of our team will be in touch with a set of requests shortly. If you do not receive a separate email within a few days, please assume that checks have been completed, and no additional changes are required.

We expect to receive your revised manuscript within 1 month. Please email us (plosbiology@plos.org) if you have any questions or concerns, or would like to request an extension.

At this stage, your manuscript remains formally under active consideration at our journal; please notify us by email if you do not intend to submit a revision so that we withdraw the manuscript.

**IMPORTANT - SUBMITTING YOUR REVISION**

Your revisions should address the specific points made by each reviewer. Please submit the following files along with your revised manuscript:

1. A 'Response to Reviewers' file - this should detail your responses to the editorial requests, present a point-by-point response to all of the reviewers' comments, and indicate the changes made to the manuscript.

*NOTE: In your point-by-point response to the reviewers, please provide the full context of each review. Do not selectively quote paragraphs or sentences to reply to. The entire set of reviewer comments should be present in full and each specific point should be responded to individually.

You should also cite any additional relevant literature that has been published since the original submission and mention any additional citations in your response.

2. In addition to a clean copy of the manuscript, please also upload a 'track-changes' version of your manuscript that specifies the edits made. This should be uploaded as a "Revised Article with Changes Highlighted " file type.

*Resubmission Checklist*

When you are ready to resubmit your revised manuscript, please refer to this resubmission checklist: https://plos.io/Biology_Checklist

To submit a revised version of your manuscript, please go to https://www.editorialmanager.com/pbiology/ and log in as an Author. Click the link labelled 'Submissions Needing Revision' where you will find your submission record.

Please make sure to read the following important policies and guidelines while preparing your revision:

*Published Peer Review*

Please note while forming your response, if your article is accepted, you may have the opportunity to make the peer review history publicly available. The record will include editor decision letters (with reviews) and your responses to reviewer comments. If eligible, we will contact you to opt in or out. Please see here for more details:

https://blogs.plos.org/plos/2019/05/plos-journals-now-open-for-published-peer-review/

*PLOS Data Policy*

Please note that as a condition of publication PLOS' data policy (http://journals.plos.org/plosbiology/s/data-availability) requires that you make available all data used to draw the conclusions arrived at in your manuscript. If you have not already done so, you must include any data used in your manuscript either in appropriate repositories, within the body of the manuscript, or as supporting information (N.B. this includes any numerical values that were used to generate graphs, histograms etc.). For an example see here: http://www.plosbiology.org/article/info%3Adoi%2F10.1371%2Fjournal.pbio.1001908#s5

*Blot and Gel Data Policy*

We require the original, uncropped and minimally adjusted images supporting all blot and gel results reported in an article's figures or Supporting Information files. We will require these files before a manuscript can be accepted so please prepare them now, if you have not already uploaded them. Please carefully read our guidelines for how to prepare and upload this data: https://journals.plos.org/plosbiology/s/figures#loc-blot-and-gel-reporting-requirements

*Protocols deposition*

To enhance the reproducibility of your results, we recommend that if applicable you deposit your laboratory protocols in protocols.io, where a protocol can be assigned its own identifier (DOI) such that it can be cited independently in the future. Additionally, PLOS ONE offers an option for publishing peer-reviewed Lab Protocol articles, which describe protocols hosted on protocols.io. Read more information on sharing protocols at https://plos.org/protocols?utm_medium=editorial-email&utm_source=authorletters&utm_campaign=protocols

Thank you again for your submission to our journal. We hope that our editorial process has been constructive thus far, and we welcome your feedback at any time. Please don't hesitate to contact us if you have any questions or comments.

Sincerely,

Melissa

Melissa Vázquez Hernández, PhD

Associate Editor

PLOS Biology

mvazquezhernandez@plos.org

on behalf of

Richard

Richard Hodge, PhD

Senior Editor

PLOS Biology

rhodge@plos.org

----------------------------------------------------------------

REVIEWERS' COMMENTS

----------------------------------------------------------------

Reviewer #1:

The authors have addressed all my comments and concerns and have gone beyond what was expected. This is now a really great piece of work. Congratulations to all involved.

----------------------------------------------------------------

Reviewer #3:

My minor comments have been addressed, but my major comments have not been addressed to a satisfactory extent. Further revisions are required, given it is an Update Article. I am not completely convinced.

1.Surprisingly, many key reports on biosensors developed for potential applications in microbiota are missing, including, but not limited to, those from the laboratory of Pam Silver, Jeff Tabor, Tim Lu, Tae Seok Moon, Harris Wang, Matthew Chang et al. In addition, key reports on the bee gut microbiome are missing, including, but not limited to, that from the Jeffrey Barrick lab. The authors should perform extensive literature search to cite and discuss relevant previous work.

�The space/reference number limitation should not be the reason for extensive literature search and detailed discussion of prior work, which is the basis for every research project. Thus, I strongly suggest that they should conduct extensive literature searches to cite and discuss more relevant previous reports.

2.I understand the challenges of developing biosensors in non-model organisms. However, the choice of sugar arabinose reduces my enthusiasm. I am less convinced regarding the utility and novelty of this work due to this choice and given that the authors developed another biosensor for IPTG in their previous report. Still, implementing this biosensor in the bee gut would be novel, but I would recommend a simple demonstration (not extensive though) of a third, more useful sensor in the bee gut because the proof-of-concept demonstration was made with the IPTG sensor.

�Demonstration of a third sensor would be beyond the scope of this work. In that case, the authors should include more extensive discussion of challenges and opportunities of developing sensors in such non-model organisms.

----------------------------------------------------------------

Reviewer #4:

I appreciate the authors' thoughtful responses, generally agree with their arguments, and believe their additions overall strengthen their manuscript.

I have only one substantial remaining concern relating to the microscopy data and analysis presented in Figures 4d, 4e, and Supplemental Figures S8 and S9. The extremely high, and highly variable, "GFP of S. alvi" in some samples in the pollen conditions (~4-10 AU) remains surprising (Fig 4D-E). Previous experiments showed a max in vivo response of ~2 AU (1000 uM arabinose- Fig 2C-G), and ~3 AU (210 uM arabinose- Fig 4C). As the methods state, the "GFP of S. alvi biofilm" is the result of normalizing the GFP intensity channel to the DAPI intensity channel, with the expectation this controls for S. alvi cell density impacting the GFP signal. This is a somewhat reasonable, it not conventional, expectation but it can also introduce error if the DAPI signal itself is variable for some technical or batch reason different than the GFP. A lower-than-average DAPI signal in particular samples, even with a commensurate GFP signal, would lead to an elevated "normalized" signal. I investigated a subset of "pollen" images "169_pollen_X_post_60X_HQ" the authors deposited into the BioImages database and found this to be the case. Several samples have a substantially lower overall DAPI signal than others, even for the eukaryotic nuclei, which should be comparable (see samples 4, 5, 7 vs 1, 2, 9). The non-pollen samples I looked at did not have this problem, with the DAPI signal overall consistent in the "ara" and "nothing" sets of 169. However, my review of the images was not comprehensive, and could be missing critical information the authors have about any batch processing or other factors. DAPI staining is notoriously variable and finicky, subject to penetrance, concentration, bleaching, and washing effects. It is often suitable to define cell locations, but not recommended to quantitatively correct for GFP.

I recommend the authors include the raw DAPI and GFP intensity values used to compute the "normalized" GFP value for all appropriate in vivo measurements as a supplemental table or supplemental figure in the manuscript. It is not clear to me that this DAPI normalization is necessary, but if the authors want to use it, they should additionally include the raw values. I attempted to use the provided FIJI macro to extract these values, but ran into technical challenges, though the macro appears sound.

# Author comment: Author response to Decision Letter 3

**DOI:** 10.1371/journal.pbio.3003997.r005

25 Aug 2026

Attachment Submitted filename: rebuttal.docx

# Editor report: Decision Letter 3

**DOI:** 10.1371/journal.pbio.3003997.r006

**Authors:** Richard Hodge

1 Sep 2026

Dear Dr Schaerli,

Thank you for the submission of your revised Update Article "An engineered bacterial symbiont maps micron-scale sugar gradients in the honeybee gut" for publication in PLOS Biology. On behalf of my colleagues and the Academic Editor, Baojun Wang, I am pleased to say that we can in principle accept your manuscript for publication, provided you address any remaining formatting and reporting issues. These will be detailed in an email you should receive within 2-3 business days from our colleagues in the journal operations team; no action is required from you until then. Please note that we will not be able to formally accept your manuscript and schedule it for publication until you have completed any requested changes.

Please take a minute to log into Editorial Manager at http://www.editorialmanager.com/pbiology/, click the "Update My Information" link at the top of the page, and update your user information to ensure an efficient production process.

PRESS

We frequently collaborate with press offices. If your institution or institutions have a press office, please notify them about your upcoming paper at this point, to enable them to help maximise its impact. If the press office is planning to promote your findings, we would be grateful if they could coordinate with biologypress@plos.org. If you have previously opted in to the early version process, we ask that you notify us immediately of any press plans so that we may opt out on your behalf.

We also ask that you take this opportunity to read our Embargo Policy regarding the discussion, promotion and media coverage of work that is yet to be published by PLOS. As your manuscript is not yet published, it is bound by the conditions of our Embargo Policy. Please be aware that this policy is in place both to ensure that any press coverage of your article is fully substantiated and to provide a direct link between such coverage and the published work. For full details of our Embargo Policy, please visit http://www.plos.org/about/media-inquiries/embargo-policy/.

Thank you again for choosing PLOS Biology for publication and supporting Open Access publishing. We look forward to publishing your study.

Sincerely,

Melissa

Melissa Vázquez Hernández, PhD

Associate Editor

PLOS Biology

mvazquezhernandez@plos.org

on behalf of

Richard Hodge, PhD, PhD

Senior Editor

PLOS Biology

rhodge@plos.org
